# The Ultrafast Quantum Dynamics of Photoexcited Adenine–Thymine Basepair Investigated with a Fragment-based Diabatization and a Linear Vibronic Coupling Model

**DOI:** 10.1021/acs.jpca.1c08132

**Published:** 2021-10-05

**Authors:** Martha Yaghoubi Jouybari, James A. Green, Roberto Improta, Fabrizio Santoro

**Affiliations:** † Consiglio Nazionale delle Ricerche, Istituto di Chimica dei Composti Organo Metallici (ICCOM-CNR), SS di Pisa, Area della Ricerca, via G. Moruzzi 1, I-56124 Pisa, Italy; ‡ Consiglio Nazionale delle Ricerche, Istituto di Biostrutture e Bioimmagini (IBB-CNR), via Mezzocannone 16, I-80136 Napoli, Italy

## Abstract

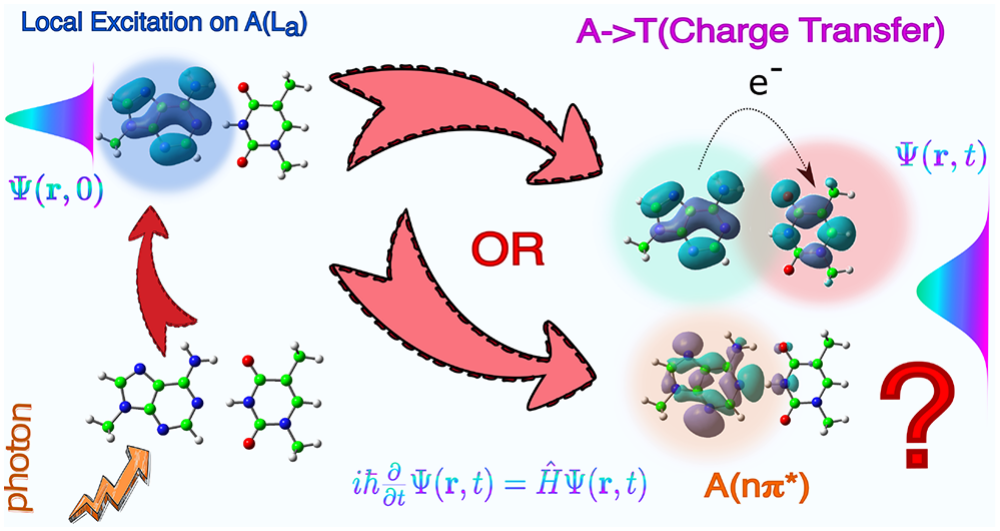

In this contribution we present a quantum dynamical study of the photoexcited hydrogen bonded base pair adenine–thymine (AT) in a Watson–Crick arrangement. To that end, we parametrize Linear Vibronic Coupling (LVC) models with Time-Dependent Density Functional Theory (TD-DFT) calculations, exploiting a fragment diabatization scheme (FrD) we have developed to define diabatic states on the basis of individual chromophores in a multichromophoric system. Wavepacket propagations were run with the multilayer extension of the Multiconfiguration Time-Dependent Hartree method. We considered excitations to the three lowest bright states, a ππ* state of thymine and two ππ* states (L_a_ and L_b_) of adenine, and we found that on the 100 fs time scale the main decay pathways involve intramonomer population transfers toward nπ* states of the same nucleobase. In AT this transfer is less effective than in the isolated nucleobases, because hydrogen bonding destabilizes the nπ* states. The population transfer to the A → T charge transfer state is negligible, making the ultrafast (femtosecond) decay through the proton coupled electron transfer mechanism unlikely, in line with experimental results in apolar solvents. The excitation energy transfer is also very small. We carefully compare the predictions of LVC Hamiltonians obtained with different sets of diabatic states, defined so to match either local states of the two separated monomers or the base pair adiabatic states in the Franck–Condon region. To that end we also extend the flexibility of the FrD-LVC approach, introducing a new strategy to define fragments diabatic states that account for the effect of the rest of the multichromohoric system through a Molecular Mechanics potential.

## Introduction

The hydrogen bonded dimer formed by adenine and thymine, especially when in its Watson–Crick (WC) arrangement, hereafter simply AT, has a fundamental biological relevance, since it is a main building block of nucleic acids. Indeed, as AT constitutes ∼60% of the human genome, in each nucleus of a human cell there are ∼0.9 billion AT pairs. Considering the critical importance of the interaction between UV light and DNA,^1−6^ which can trigger many potentially harmful oxidative processes,^7,8^ the photoactivated dynamics of AT has been the object of several studies, both experimental^9,10^ and computational,^11−16^ without considering those discussing its behavior when inserted in a duplex.^3^ In a seminal contribution Perun, Soboloweski, and Domcke^11^ suggested that in the gas phase a possible excited-state deactivation mechanism for a WC AT pair involves a Proton Coupled Electron Transfer (PCET) process. In the proximity of their minima, the bright local excitations (LEs) on A or on T can cross the lowest energy A → T charge transfer (CT) state, A → T CT, which is significantly less stable (by ∼1 eV) in the Franck–Condon (FC) region. A small increase of one of the amino N–H bond lengths is sufficient to activate this crossing, while proton transfer to T strongly stabilizes the A → T CT and leads, without any energy barrier, to a conical intersection with S_0_. This mechanism, operative only for a WC arrangement, is very similar to that described for the guanine–cytosine (GC) pair, which has been shown to occur also in the gas phase^15,17−24^ and, very likely, in chloroform solution.^25^ This picture was basically confirmed by more recent computational studies: that PCET is a possible deactivation route for the A → T CT in the gas phase,^12−14^ in water,^12^ and also in DNA duplex.^26^ However, the studies based on the static exploration of the potential energy surface (PES) have also confirmed that the population of the A → T CT state, though in principle feasible, is expected to be more difficult than that of the corresponding G → C CT state, whose stability is similar to that of the bright excited states localized on G and C. Furthermore, recent experiments have shown that PCET does not play a significant role in the photoexcited dynamics of WC AT pair in chloroform.^9^

The formation of WC pair, besides making the A → T CT state possible, can have additional more subtle, but not less significant, consequences on the local excited states of A and T. For both individual bases, dark excited states with nπ* character, involving the lone pair of nitrogen atoms of A or carbonyl oxygen atoms of T, are important players in excited-state dynamics, especially in the gas phase.^27−45^ However, the involvement of the nπ* states is likely to be altered in the WC pair, due to hydrogen bonding (HB) interactions causing their destabilization.^46^ Finally, HB could also affect the interplay between the two lowest bright excited states of A, usually labeled as L_a_ and L_b_, whose relative stability has been matter of debate in the isolated nucleobase.^3,47,48^

In this work we perform the first quantum dynamics (QD) study on the photoexcited AT WC pair in the gas phase, using our recently developed fragment diabatization linear vibronic coupling (FrD-LVC) methodology,^49^ in combination with the multilayer multiconfiguration time-dependent Hartree (ML-MCTDH) method.^50−52^ Quantum nuclear effects are indeed expected to be important in processes like the ones we study here, with several coupled states lying at similar energies.

We investigate the electronic population dynamics following initial excitation of the lowest ππ* state of T and the L_a_ and L_b_ states of A. The usage of LVC model Hamiltonians prevented us to include the direct monomer-like pathways to the ground state, since they occur at geometries with large amplitude displacement, limiting the relevance of the obtained results for AT photophysics to the first ∼100 fs after photoexcitation, when large distortions of the molecular structures out of planarity are unlikely. This time scale is, however, sufficient to provide two interesting indications on the photophysics of AT: (i) nπ* states are populated for both T and A, albeit to a lesser extent than for the individual nucleobases we have recently investigated with a similar methodology,^42^ and (ii) no transfer to the A → T CT state is predicted.

From the methodological point of view, we investigate the impact of different definitions of the diabatic states on the predictions of absorption spectra and photoinduced dynamics. In particular, we choose as reference states either the adiabatic states of AT at the ground-state equilibrium geometry (standard approach) or local states on the fragments A and T. For the latter case, we introduce here an alternative procedure to determine the reference states in addition to that which we have presented previously,^49^ where the effect of the second base may be considered at molecular mechanics (MM) level. This permits us to analyze some issues of general relevance for the study of Multi-Chromophore (MC) assemblies, such as the dependence of simulated dynamics on the choice of the reference states and on the extension of the diabatic basis set, presenting results with all-coordinates models (102 normal modes) including from 12 up to 32 electronic states.

## Methods

In this work, we parametrize a LVC model for MC systems made up of *N*_frag_ fragments/chromophores. The form of the LVC Hamiltonian for a set of *N* coupled diabatic electronic states |*d*_*i*_⟩ (*i* = 1, ..., *N*) is
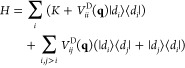
1where **q** are the dimensionless normal mode coordinates of the ground electronic state S_0_ and **p** are the conjugate momenta. The expressions for the kinetic *K* and potential *V* terms are
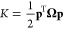
2
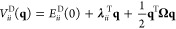
3
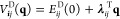
4Here *E*_*ii*_^D^(0) and *E*_*ij*_^D^(0) are the diabatic energy of state *i* and the electronic coupling constant between diabatic states *i* and *j* at the reference geometry (0), **Ω** is the diagonal matrix of the S_0_ normal-mode frequencies, and **λ**_*ii*_ and **λ**_*ij*_ (*j* ≠ *i*) are the vectors of the energy gradients and linear coupling constants. Notice that the *E*_*ij*_^D^(0) terms do not appear in the standard description of the LVC approach^53^ but become necessary when the diabatic states are not coincident with the adiabatic ones at the reference geometry.

We define the transformation from the adiabatic to diabatic basis using Löwdin orthogonalization
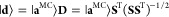
5where **S** is defined as the overlap matrix between reference states and adiabatic states of the MC with elements *S*_*im*_ = ⟨*R*_*i*_|*a*_*m*_^MC^⟩ (N.B. throughout we use the indices *i*, *j* for diabatic states and *m*, *n* for adiabatic states). The transformation yields diabatic states that are the combination of adiabatic states that resemble as much as possible the reference states. We consider 3 different choices of reference states in order to examine their effect on the LVC model, and resultant dynamics.

1. We set the reference states as equivalent to the adiabatic states of the MC at the reference geometry and label this as the standard LVC (St-LVC) approach. This is a typical choice adopted for LVC models, and we have used this method previously in the study of individual chromophores.^42,54−58^
2. We use our recently proposed fragment diabatization (FrD) technique to define the reference states.^49^ In this approach, the reference states are the adiabatic states of the isolated fragments of the MC (for LEs), or one electron transitions between orbitals on different fragments (for CT states). This permits a definition of the diabatic states in terms of an excitonic model-like individual site basis and can lead to a more chemically intuitive interpretation than the St-LVC approach, if adiabatic states of the MC are somewhat delocalized. We label this approach FrD-LVC.
3. We propose a different implementation of the FrD-LVC approach, in which the reference states are still defined by calculations on the individual fragments, which however include the effects of the surrounding fragments in a MM fashion. In this way we retain the intuitive individual site basis, but account for the change in LE character and orbital shape due to the electrostatic effects of the surroundings. In the following the calculations adopting this strategy will be labeled FrD(MM_ref_)-LVC.

An illustration of these choices is shown in Figure S1 of the Supporting Information (SI). Using these definitions of the reference states, we can compute the parameters of the LVC model in eqs 3 and 4. For the St-LVC model, *E*_*ii*_^*D*^(0) are simply equal to the adiabatic energies of the MC and *E*_*ij*_^D^(0) are equal to 0, while for both FrD-LVC approaches, we perform the transformation in eq 5 at the reference geometry, to yield the transformation matrix **D**(0), which can be applied to the diagonal matrix of adiabatic energies of the MC to obtain *E*_*ii*_^D^(0) and *E*_*ij*_^D^(0) parameters.

To obtain the linear coupling constants **λ**_*ij*_, we displace each normal coordinate α of the MC by some small values ±Δ_α_, find a new transformation matrix **D**(±Δ_α_), and perform a numerical differentiation.^49^

As previously done for DNA nucleobases and G-quadruplexes,^42,54−56,59^ we express the reference states and overlap matrix within the framework of TD-DFT, and further details can be found in these papers. Similarities and differences of our approach with the work of Tamura and Burghardt on conjugated polymers and fullerene systems^60−68^ have also been analyzed in ref (49).

## Computational Details

Electronic structure calculations have been performed with DFT for the ground state and TD-DFT for the excited states using the Gaussian 16 program.^69^ We adopted the CAM-B3LYP^70^ range-separated functional, previously validated for the study of AT,^71^ and the computationally convenient 6-31G(d) basis set. For the parametrization of the LVC Hamiltonian, TD-DFT computations were performed using tight SCF convergence and a 10^–6^ au threshold for the energy (the same as recommended for taking numerical derivatives of the energy).

**Figure 1:**
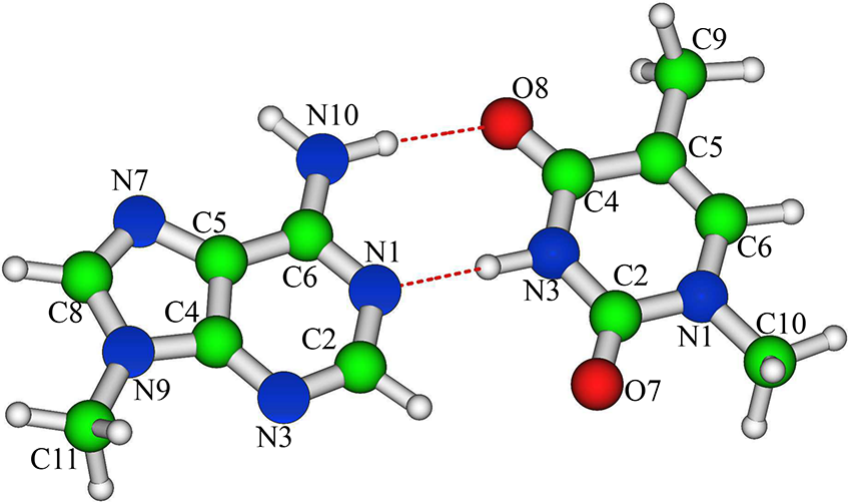
Schematic drawing and atom labeling of the computational model of the 9-methyladenine and 1-methylthymine dimer in a Watson–Crick arrangement. Sugar rings are modeled by the methyl groups bonded at adenine-N9 and thymine-N1.

We consider a molecular model of the WC base pair of adenosine and thymidine replacing the sugars with methyl groups, obtaining 9-methyladenine and 1-methylthymine held together by two hydrogen bonds as in Figure 1. Ground- and excited-state geometries were optimized with *C*_*s*_ symmetry, since this permits the decoupling of the A′ (ππ* and CT) and A″ (nπ*) states. Nuclear motion is described using the normal modes of the dimer, since this naturally allows the investigation of the effect of intermolecular vibrations. According to the recipe of the LVC model, the vibrational frequencies computed for the S_0_ state are then utilized also for each of the diabatic excited states. For the FrD(MM_ref_)-LVC calculations, reference local states on each nucleobase were computed describing the electrostatic effect of the other nucleobase by the set of the RESP charges for its ground electronic state. The same molecular orbitals obtained in these QM/MM calculations were adopted to define CT diabatic states, and among them the most relevant is the orbital transition from the HOMO of A (HOMO_A_) to the LUMO of T (LUMO_T_).

We parametrized LVC models with different numbers of states, and in all cases, to ensure a full projection of the reference diabatic states, we adopted a large set of 40 adiabatic states of AT computed with TD-DFT (see eq 5). The diabatization was performed with an in-house code interfaced with Gaussian 16 that is freely available upon request. LVC models for individual 9-methyladenine and 1-methylthymine have also been parametrized at the same level of theory following the protocol for individual bases we have recently used.^42,54,72^ Since in the diabatization procedure we compute the projection of the diabatic states onto the adiabatic states of the AT dimer, this information can be employed to precisely measure the similarity among diabatic and adiabatic states. In more detail, for each adiabatic state *m* we can compute the square of the elements of the overlap matrix *S*_*im*_ = ⟨*R*_*i*_|*a*_*m*_^MC^⟩ for each of the *N* reference states *i*. Then, we define the weight of the reference state with maximal overlap with the adiabatic state as *W*_*m*_ = max_*i*∈*N*_ *S*_*im*_^2^.

ML-MCTDH wave packet propagations^50−52^ were performed with the Quantics package,^73,74^ using a variable mean field (VMF) with a RungeKutta integrator of order 5 and accuracy 10^–7^, as in previous applications to other DNA nucleobases^42,54,72^ and the GC base pair.^49^ For the primitive basis set, we adopted Hermite DVR functions. Convergence of the QD propagations was checked by monitoring the populations at the beginning and end of the grid using the rdgpop tool provided in Quantics, ensuring that they did not exceed 10^–9^. For the ML “tree” expansions (reported in the SI), we chose the number of single particle functions (SPFs) for each node based on the magnitude of the linear coupling constants λ_*ii*,α_, with modes with larger couplings assigned larger numbers of SPFs, as we have done in recent studies of single nucleobases.^42,54−56,72^ Further convergence checks were done by monitoring the eigenvalues of the density matrices of each node in the ML tree, ensuring that the smallest natural weight was always <1% as indicated in the Quantics manual. Finally, convergence was also confirmed by changing the number of SPFs and the dimension of the primitive basis set. Some of these tests are shown in section S2 of the SI together with a graphical representation of the ML-MCTDH trees. Since our LVC Hamiltonians do not account for monomer-like decays to the ground state, the relevance of their predictions for the AT physics decreases after the first ∼100 fs. We, however, report the time-dependent populations up to 250 fs, since in many other applications LVC models are reliable also for longer times and therefore it is still of interest to analyze the dependence of results on different LVC parametrizations.

## Results and Discussion

### The Excited States at the FC Position

**Figure 2:**
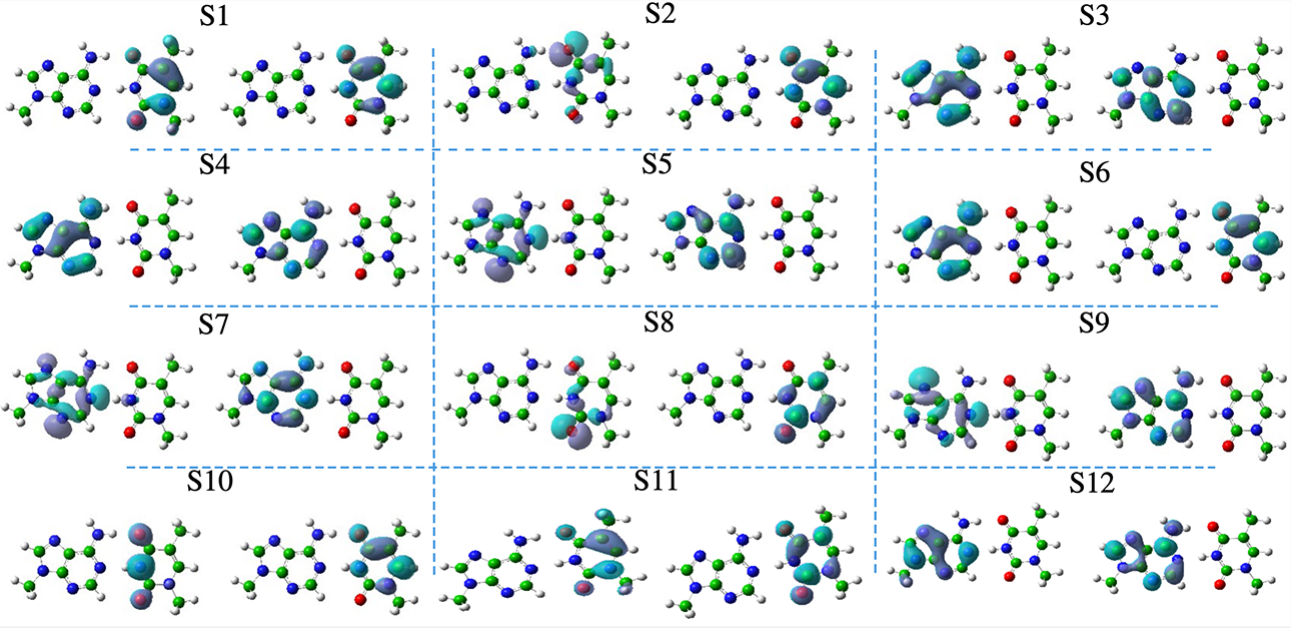
NTOs of first 12 states of AT in the gas phase at the ground-state geometry *C*_*s*_ symmetry computed at the CAM-B3LYP/6-31G(d) level of theory with an isovalue of 0.04.

In Table 1 we give a concise description of the lowest energy excited states in the ground-state minimum (FC point). Their representation in terms of natural transition orbitals (NTOs) is given in Figure 2, while the corresponding Kohn Sham (KS) molecular orbitals are sketched in Figure S4 of the SI. Although the excited states of AT are in principle delocalized over the two bases, Figure 2 indicates that, for the first 12 excited states, it is qualitatively possible to establish a one-to-one correspondence with excited states of isolated A and T, and the A → T CT state is also readily identifiable. In fact, S_1_ is a ππ* on thymine; S_3_ and S_4_ two ππ* on adenine (respectively L_a_ and L_b_, but we discuss this further in the next subsection); S_6_ is a A → T CT state (from HOMO_*A*_ to LUMO_*T*_); S_2_ and S_8_ are two nπ* of thymine; S_5_, S_7_, and S_9_ are three nπ* of adenine; and finally S_10_ and S_11_ are a second and third ππ* on thymine and S_12_ a third ππ* on adenine. This (quasi-)localized description is in contrast with what we observed previously for GC, where there was significant delocalization and mixing of the states, in particular the ππ* and CT ones.^49^

**Table 1:** Symmetry (Sym), Electronic Characters, TD-DFT Energies (*E*_*m*_^A,DFT^, in eV, with Respect to S_0_ at the FC Point), Weight of the Predominant Diabatic State in the Adiabatic State for Both FrD(MM_ref_)-LVC and FrD-LVC (*W*_RESP_, *W*_isolated_), and Oscillator Strengths δ_OPA_, Computed for AT at the FC Point^a^

state S_*m*_	sym	character	*E*_*m*_^A,DFT^	*W*_RESP_	*W*_isolated_	δ_OPA_	CC2^11^
S_1_	A′	T(ππ*1)	5.32	0.96	0.95	0.204	5.37 (S_3_)
S_2_	A″	T(n_O_π*1)	5.36	0.99	0.99	0.000	5.13 (S_1_)
S_3_	A′	A(L_a_)	5.45	0.98	0.94	0.126	5.25 (S_2_)
S_4_	A′	A(L_b_)	5.56	0.95	0.93	0.203	5.45 (S_4_)
S_5_	A″	A(n_N_π*1)	5.66	0.99	0.91	0.000	5.51 (S_5_)
S_6_	A′	A → T (CT)	6.08	0.97	0.97	0.003	6.26 (S_7_)
S_7_	A″	A(n_N_π*2)	6.14	0.99	0.96	0.000	6.03 (S_6_)
S_8_	A″	T(n_O_π*2)	6.44	0.99	0.99	0.000	6.31 (S_8_)
S_9_	A″	A(n_N_π*3)	6.61	0.99	0.91	0.003	
S_10_	A′	T(ππ*2)	6.63	0.85	0.85	0.017	6.64 (S_10_)
S_11_	A′	T(ππ*3)	6.73	0.84	0.83	0.296	6.91 (S_12_)
S_12_	A′	A(ππ*3)	6.81	0.71	0.71	0.268	6.82 (S_11_)

a CAM-B3LYP/6-31G(d) calculations. Also shown for comparison are CC2/cc-pVDZ energies and state ordering from ref (11). Additional TD-DFT data are reported in Table S1 in the SI.

Also shown for comparison are the CC2/cc-pVDZ vertical absorption energies from ref (11). The energies and ordering of the states are predominantly similar to those of CAM-B3LYP/6-31G(d). The main discrepancy concerns the energy and position of the T(n_O_π*1) state, which is predicted to be the most stable state by CC2 and 0.24 eV more stable than the T(ππ*1) state, whereas CAM-B3LYP predicts them to lie at more similar energies. However, the trend of destabilization of the T(n_O_π*1) state in the AT pair relative to the isolated base is reproduced, with CC2/cc-pVDZ predicting the n_O_π*1 state to be 0.46 eV more stable than ππ*1 in isolated T^75^ and CAM-B3LYP/6-31G(d) predicting it to be 0.36 eV more stable.^42^

We are interested in studying the nonadiabatic dynamics after a photoexcitation to the first three lowest bright states, investigating the possible involvement of nπ* and CT states. Therefore, in order to build up our FrD-LVC Hamiltonian we consider 12 reference (local and CT) states with the same characters as the 12 TD-DFT adiabatic states at the FC point, as previously discussed. Although we do not investigate the dynamics following photoexcitation to the higher lying ππ* states (i.e., T(ππ*2), T(ππ*3), and A(ππ*3)), we include them in the FrD-LVC models as they are at similar energy to the highest nπ* states and/or are strongly electronically coupled with the lower lying ππ* states (see Table S4 in the SI). These 12 reference states were computed for the fragments held exactly in the same position as in the base pair with the two different strategies, i.e., FrD and FrD(MM_ref_). For comparison we also parametrized a standard LVC Hamiltonian (St-LVC), defining 12 diabatic states that coincide with the 12 adiabatic states of AT in the FC position.

Although qualitatively similar, the diabatic states of the three Hamiltonians are not formally identical (see Figure S1 in the SI). A deeper analysis is possible by computing the weights defined in the Computational Details. They are given in columns 5 and 6 in Table 1 and labeled as *W*_RESP_ and *W*_isolated_ for FrD(MM_ref_) and FrD, respectively. These values are mostly ≥0.9, justifying the use of the same labels for corresponding adiabatic/diabatic states.

Furthermore, as shown in Table 2, at the FC point the energies of the FrD and FrD(MM_ref_) diabatic states (*E*_*ii*_^D^) are very close to the TD-DFT energies of the corresponding adiabatic states (*E*_*m*_^A,DFT^). The largest deviation is observed for the A → T (CT) which is predicted to be 0.06 eV (0.08 eV) higher in energy according to FrD(MM_ref_) (FrD) strategies. In general, when compared to FrD, FrD(MM_ref_) provides diabatic energies closer to the TD-DFT ones and larger *W*_RESP_ overlaps. This outcome confirms that the FrD(MM_ref_) strategy, accounting for some effects of the presence of the other nucleobase, allows defining fragment diabatic states closer to the TD-DFT ones of the dimer.

**Table 2:** Energies (eV) of the Diabatic States (*E*_*ii*_^D^) from Different Diabatization 12-State Models, Compared with the Adiabatic Energies with the Same Predominant Character Obtained with TD-DFT (*E*_*m*_^A,DFT^) and via Diagonalization of the LVC Hamiltonians (*E*_*m*_^A,LVC^) and the TD-DFT Adiabatic Energies of the Local Excitations for the Isolated Single Bases or the Single Bases in the Presence of the RESP Charges of the Other Base^a^

		base pair					single base	
		TD-DFT	FrD(MM_ref_)		FrD		TD-DFT	
							MM_ref_	isolated
ad. state *m*/diab state *i*	character	*E*_*m*_^A,DFT^	*E*_*ii*_^D^	*E*_*m*_^A,LVC^	*E*_*ii*_^D^	*E*_*m*_^A,LVC^	*E*_*m*_^A,DFT^	*E*_*m*_^A,DFT^
1	T(ππ*1)	5.32	5.34	5.33	5.35	5.33	5.35	5.39
2	T(n_O_π*1)	5.36	5.41	5.41	5.42	5.41	5.29	5.17
3	A(L_a_)	5.45	5.46	5.46	5.48	5.46	5.50	5.51
4	A(L_b_)	5.56	5.57	5.57	5.58	5.57	5.59	5.60
5	A(n_N_π*1)	5.66	5.68	5.67	5.76	5.68	5.64	5.45
6	A → T (CT)	6.08	6.14	6.14	6.16	6.16		
7	A(n_N_π*2)	6.14	6.15	6.15	6.20	6.21	6.16	6.05
8	T(n_O_π*2)	6.44	6.44	6.44	6.45	6.45	6.40	6.44
9	A(n_N_π*3)	6.61	6.62	6.63	6.59	6.65	6.59	6.42
10	T(ππ*2)	6.63	6.66	6.64	6.66	6.66	6.75	6.83
11	T(ππ*3)	6.73	6.74	6.73	6.74	6.73	6.71	6.66
12	A(ππ*3)	6.81	6.77	6.81	6.76	6.82	6.78	6.76

a Calculated at the equilibrium geometry of AT in *C*_*s*_ symmetry by CAM-B3LYP/6-31G(d).

The so-called L_a_ and L_b_ states of A deserve special attention. In isolated A at its ground-state geometry L_a_ is the stronger absorbing state with a dominant H → L character. At the TD-DFT level of theory L_a_ is also more stable than L_b_, which is the weaker absorbing state, with a dominant H → L+1 character.^3^ For isolated A in the AT geometry the two configurations are very mixed at the CAM-B3LYP/6-31G(d) level of theory, although the H → L transition is still dominant in the lower energy state (see Figure S5 in the SI).

Inspection of Figure 2 and Table S1 and Figures S4 and S5 in the SI show that in AT S_3_ and S_4_ are clearly associated with the L_a_ and L_b_ states of A. In terms of whether L_a_ or L_b_ correspond to S_3_ or S_4_, S_3_ is weaker and its main contribution in terms of the KS orbitals (Figure S5 in the SI) looks similar to the H → L+1 transition of A. Although this finding might suggest a switching of the order of the states in the base pair, the weights in Table 1 and a careful analysis in the SI, section S3.1.1, show that S_3_ of AT has to be assigned to L_a_ and S_4_ to L_b_, since what really discriminates the two states is the in-phase/out-of-phase combination of the orbital transitions. In any case, these two states also exhibit some small components from the other state of adenine (L_b_ or L_a_) and even from the lowest ππ* of T (see Table S6 in the SI).

Diagonalization of the LVC Hamiltonian at the FC position (**q** = **0**) allows us to compute LVC adiabatic energies (*E*_*m*_^A,LVC^), also reported in Table 2. They are similar to the diabatic ones but, as may be expected, generally closer to the TD-DFT adiabatic ones at least for the low-lying states. In Tables S2 and S3 of the SI the full matrices of the LVC adiabatic eigenvectors at the FC position are reported (separated by symmetry), while Tables S4 and S5 report the corresponding A′ and A″ blocks of the full diabatic Hamiltonian matrices. It is worthy to recall that the adiabatic LVC energies *E*_*m*_^A,LVC^ are not expected to be identical to the adiabatic TD-DFT ones *E*_*m*_^A,DFT^, since the fragment diabatic basis set is not, in general, complete. Moreover, the LVC diabatic energies *E*_*ii*_^D^, are different from the adiabatic ones *E*_*m*_^A,LVC^ due to the nonzero couplings *E*_*ij*_^D^.

The last columns of Table 2 give the TD-DFT energies of the references states obtained in the calculation of the fragments. Although similar to the diabatic energies of the dimer they are not identical. When compared to the TD-DFT adiabatic energies of the dimer, the isolated bases show a ∼0.2 eV red shift of the nπ* states, whereas when the other base is included in an MM fashion, this red shift is much smaller, suggesting that a significant part of the HB effect is already captured at the classical level.

### Excited-State Minima

Table 3 reports the energy of the 12 diabatic states (provided by the different procedures) in their minima, together with the closest LVC adiabatic state (obtained by the diagonalization of the LVC potential-energy matrix). The corresponding eigenvalues and eigenvectors are given in Tables S8 and S9 in the SI, separated by symmetry.

**Table 3:** LVC Energies (eV) of the Diabatic (*E*_*ii*_^D^[*D*_min_]) and Adiabatic (*E*_*m*_^A,LVC^[*D*_min_]) States of AT at the Diabatic State Minima (*D*_min_) Predicted by the Different LVC Models and Selected TD-DFT Adiabatic Energies at the TD-DFT Predicted Adiabatic Minima *E*_*m*_^A,DFT^[*A*_min_]^a^

		St-LVC		FrD(MM_ref_)-LVC		FrD-LVC		TD-DFT
ad. state *m*/diab state *i*	character	*E*_*ii*_^D^[*D*_min_]	*E*_*m*_^A,LVC^[*D*_min_]	*E*_*ii*_^D^[*D*_min_]	*E*_*m*_^A,LVC^[*D*_min_]	*E*_*ii*_^D^[*D*_min_]	*E*_*m*_^A,LVC^[*D*_min_]	*E*_*m*_^A,DFT^[*A*_min_]
1	T(ππ*1)	4.99	4.98 (0.98)	4.99	4.99 (0.99)	5.00	5.00 (0.99)	4.99
2	T(n_O_π*1)	4.97	4.96 (0.99)	4.96	4.96 (0.99)	4.97	4.97 (0.99)	4.85
3	A(L_a_)	5.16	5.11 (0.94)	5.17	5.12 (0.94)	5.19	5.14 (0.93)	5.08
4	A(L_b_)	5.33	5.27 (0.67)	5.33	5.29 (0.67)	5.36	5.30 (0.56)	
5	A(n_N_π*1)	5.08	5.06 (0.98)	5.12	5.10 (0.98)	5.21	5.19 (0.98)	4.99
6	A → T (CT)	5.00	4.93 (0.95)	4.99	4.99 (0.99)	5.01	5.01 (0.99)	4.82
7	A(n_N_π*2)	5.66	5.73 (0.58)	5.65	5.73 (0.54)	5.74	5.80 (0.62)	
8	T(n_O_π*2)	5.86	5.90 (0.64)	5.86	5.80 (0.55)	5.89	5.94 (0.62)	
9	A(n_N_π*3)	6.24	6.23 (0.80)	6.25	6.25 (0.84)	6.23	6.25 (0.96)	
10	T(ππ*2)	6.14	5.99 (0.57)	5.96	5.96 (0.98)	5.97	5.94 (0.97)	
11	T(ππ*3)	6.34	6.21 (0.87)	6.20	6.19 (0.99)	6.22	6.20 (0.98)	
12	A(ππ*3)	6.70	6.75 (0.47)	6.61	6.62 (0.99)	6.62	6.62 (0.99)	

a Values in parentheses give the weight of the corresponding diabatic state in the adiabatic state. LVC models are parameterized with CAM-B3LYP/6-31G(d) calculations.

The three diabatization procedures predict that the two minima with lowest diabatic energy are T(n_O_π*1) and T(ππ*1), with the former being slightly more stable. The minimum of the A → T CT state is then practically degenerate with T(ππ*1), and actually, according to St-LVC, its adiabatic energy is 0.03 eV lower than that of T(n_O_π*1). We then find three minima localized on A: A(n_N_π*1), A(L_a_), and ∼0.17 eV diabatically higher in energy, A(L_b_).

Interestingly, recomputing the TD-DFT states at these diabatic minima and looking at main contributions in terms of transitions among MOs (see Figure S6 in the SI), the electronic character of the adiabatic states labeled L_a_ and L_b_ becomes more pure; i.e., the states show a greater contribution from HOMO → LUMO or HOMO → LUMO+1 of the fragment. Of course, since these two excitations contribute to both the L_a_ and L_b_ diabatic states defined at the FC position, such behavior in the minima is reflected, from the point of view of the LVC states, in a substantial mixing of the L_a_ and L_b_ in the LVC adiabatic states (see Table S8 in the SI).

The energy of the A(n_N_π*1) minimum is the one depending the most on the diabatization procedure. It lies at 5.08 eV according to St-LVC, at 5.12 eV according to FrD(MM_ref_), and at 5.21 eV according to FrD. Consequently, according to St-LVC and FrD(MM_ref_), the most stable minimum localized on A is the A(n_N_π*1) minimum, while according to FrD it is the A(L_a_) minimum (see Table S10 in the SI). As we will discuss below, this difference will affect the QD simulations.

In section S3.2 of the SI we further investigate the stability of A(L_a_), A(L_b_), and A(n_N_π*1) since, as shown in the following, different models can predict remarkably different populations. At the diabatic minima, LVC adiabatic energies are quite similar to TD-DFT ones recomputed at the same geometries, the largest deviation being for A(n_N_π*1) which is 0.09 eV more stable according to TD-DFT (see Figure S6 and Table S9 in the SI). We also attempted TD-DFT optimizations of these states starting from the diabatic minima geometries, and these TD-DFT minima are labeled as *E*_*m*_^A,DFT^(*A*_min_) in Table 3. We located A(L_a_) and A(n_N_π*1) TD-DFT planar minima and they exhibit only a very slight stabilization (∼0.03 eV) indicating that the minimum geometries estimated by LVC are quite accurate. The optimization algorithm failed to optimize A(L_b_), ending again in the A(L_a_) minimum.

Starting from the corresponding diabatic minimum, we also successfully optimized the A → T CT state, keeping the N10–H bond length on A fixed in order to prevent proton transfer and permit best comparison to the LVC model. We found that it lies at 4.82 eV, being somewhat more stable than those estimated by the LVC models (see Table 3), due both to moderate structural differences and differences in the hydrogen bonding lengths. We intend to study the proton transfer in a future work, and in this respect it is worthwhile to note that previous studies have shown that CAM-B3LYP^13^ and M05-2X^12^ functionals give similar energetics to the CC2 results of Perun et al.^11^ along this coordinate.

### Absorption Spectrum

**Figure 3:**
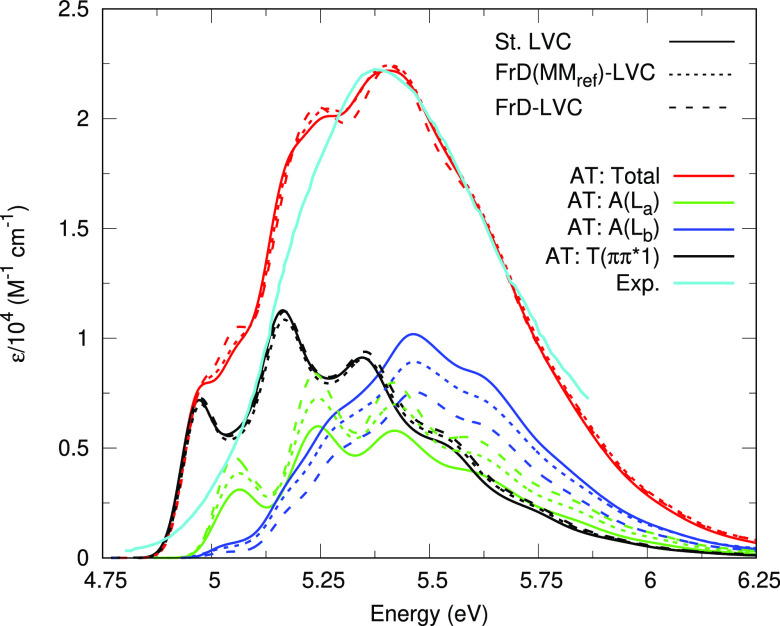
Absorption spectra of AT (red) calculated using the standard LVC (solid), FrD(MM_ref_)-LVC (dotted), and FrD-LVC (dashed) 12-state models, showing the contribution from the propagations on the three bright states, A(L_a_) (green), A(L_b_) (blue), and T(ππ*1) (black), and compared with an experimental spectrum of a modified AT WC pair in chloroform (cyan).^9^ Experimental spectrum blue-shifted by 0.7 eV and calculated spectra broadened with Gaussians of half-width-half-maxima of 0.04 eV.

We calculated the nonadiabatic absorption spectrum of AT in the gas phase following photoexcitation to each of the lowest 3 bright states, i.e., T(ππ*1), A(L_a_), and A(L_b_). This is shown in Figure 3 and is compared to the experimental spectrum of a substituted AT base pair measured in chloroform.^9^ To calculate the spectrum, we follow the procedure we have used recently for GC^49^ and individual nucleobases.^42^ Further details may be found in the SI, section S1.2. The computed spectrum shows a large blue shift of ∼0.7 eV with respect to the experiment. In ref (49) we applied the same protocol to compute the spectra of all the 5 isolated DNA and RNA nucleobases in gas phase finding a blue shift of ∼0.45–0.5 eV. Therefore, it is likely that the additional ∼0.2 eV, found here, is due to the lack of solvent contributions. The agreement for the spectral shape is generally good, although the computed spectrum shows some residual vibronic structure (our phenomenological broadening may be too narrow to reproduce solvent broadening) and is slightly too broad, probably indicating some inaccuracy in the relative vertical transitions of A and T. Figure 3 also reports the contributions to the total spectra due to propagations initiated on the three bright states A(L_a_), A(L_b_), and T(ππ*1) (their sum gives the total spectrum^49^). Interestingly, the contributions of the dynamics on A(L_a_) and A(L_b_) show appreciable differences in the three models, due to the differences in the definition of the two states. However, the total spectra are extremely similar confirming the robustness of our results. The contribution to the spectrum arising from the dynamics started on T(ππ*1) is also very similar in the three models.

In the following sections we study the dynamics of the electronic populations after a photoexcitation to the lowest bright state of T, T(ππ*1), and the two lowest bright states of A, A(L_a_), and A(L_b_). At our level of calculation this means an excitation up to ≤5.5 eV. Considering the computational error discussed above, these simulations should cover most of the processes triggered by light absorption up to 260 nm (∼4.8 eV).

### Quantum Dynamics of the Electronic Populations

**Figure 4:**
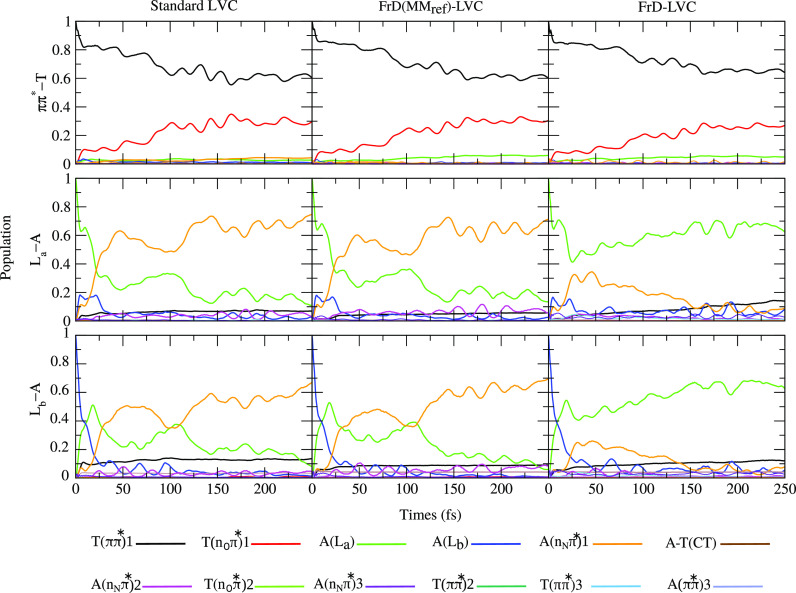
Diabatic-state populations for AT with initial excitation of T(ππ*1) (top), A(L_a_) (middle), and A(L_b_) (bottom) obtained with the standard LVC (left), FrD(MM_ref_)-LVC (middle), or FrD-LVC (right) models.

In Figure 4 we report the prediction of the three LVC models St-LVC (left), FrD(MM_ref_)-LVC (center), and FrD-LVC (right) for the time evolution of the electronic populations.

As clearly shown by Figure 4, our QD simulations predict that, independently of the adopted LVC model, both T and A in the AT base pair mostly undergo intramolecular decays from the bright ππ* to the dark nπ* states localized on the same photoexcited base (T or A). This is a first important indication: the intermonomer decay paths do not play a significant role in the AT photoactivated dynamics. In fact, as discussed further below, the population transfer to A → T CT is very small. Also excitation energy transfers are very limited, as shown by the 10% population of T(ππ*1) after excitation of L_a_ or L_b_.

In detail, all three LVC models provide convergent results for an initial photoexcitation to T(ππ*1), predicting a population of T(n_O_π*1) at *t* = 100 fs of ∼30% and a long-time limit population of ∼40%. This population transfer is strongly reduced with respect to isolated T, as shown in the SI, Figure S8. For this system, our simulations, parametrized at the same level of theory, predict that for an excitation to T(ππ*1), T(n_O_π*1) acquires ∼80% of the population in 100 fs and ∼90% in 200 fs.

For initial excitation to A(L_a_) in AT, after 25 fs 40% of the population has been transferred to A(n_N_π*1). The dynamics at longer times then depend on the adopted LVC model. FrD-LVC predicts that the population of the dark-state A(n_N_π*1) is only transient, lowering to <20% at *t* = 100 fs and to <10% at *t* > 150 fs; on the contrary, according to St-LVC and FrD(MM_ref_)-LVC the population of A(n_N_π*1) is ∼35% at *t* = 100 fs and persists at longer times, even increasing up to ∼70%. This discrepancy is mainly related to the relative stability of A(L_a_) and A(n_N_π*1). As discussed above, the A(L_a_) minimum is less stable than the A(n_N_π*1) minimum according to St-LVC and FrD(MM_ref_)-LVC, whereas the former is more stable according to FrD-LVC. The exact determination of the partial population on A(n_N_π*1) in the long-time limit revealed to be quite challenging, and it is further discussed in the following section, since it gives rise to interesting methodological issues. The prediction of a LVC model on that time scale has, however, a limited interest for AT photophysics. In fact, we recall that previous studies on A photoactivated dynamics have shown that for nonplanar structures A(L_a_) and A(n_N_π*1) are strongly coupled.^3,76^ Notwithstanding this, the two states are expected to preferentially follow two different nonradiative decay paths, involving out-of-plane distortion of the C2 (for L_a_) or the C6-NH_2_ groups (n_N_π*1). It is clear that this complex photophysics cannot be described by LVC calculations, although they do confirm the strong vibronic coupling between these two states.

When exciting A(L_b_), the population first flows to A(L_a_) which acts as a doorway to A(n_N_π*1) in all models. This is in line with the fact that A(L_b_) and A(L_a_) are strongly vibronically coupled and that A(n_N_π*1) is more coupled to A(L_a_) than to A(L_b_) (see Table S7 in the SI).

On balance, independently of the discrepancies between the different LVC models, they agree that WC pairing partially quenches the population transfer to A(n_N_π*1), similar to what was observed for T. Indeed, for isolated A, Figure S8 in the SI shows that after exciting either A(L_a_) or A(L_b_), A(n_N_π*1) reaches a population of ∼90% in ∼100 fs. These results on A are in agreement with what is already shown for CAM-B3LYP and different basis sets in ref (42).

Another clear indication from the plots of Figure 4 is that exciting the ππ* states on either T or A, the A → T CT state is not populated. This is a marked difference with what is predicted for the GC base pair,^49^ suggesting that for the AT base pair the PCET decay mechanism of Domcke and Sobolewski^17,18^ should not be operative in the ultrafast regime. One important difference between AT and GC obviously concerns the relative stability of the CT state in the FC point. A → T CT is ∼0.7 eV less stable than A(L_a_) and ∼0.6 than A(L_b_), while with a similar level of theory the G → C CT is predicted to be ∼0.25 eV more stable than G(L_a_).^49^ Although the CT state has a very large reorganization energy (∼1.2 eV in both GC^49^ and AT), due to the different stability in the FC region, in GC its minimum is by far the most stable, whereas in AT, its minimum is practically degenerate to the T(ππ*1). However, G → C CT is populated, even if only slightly, also when it is shifted in the FC region so to be 0.6 eV less stable than G(L_a_).^49^ This suggests that other effects are operative (see additional data in section S3.3.2 in the SI). In particular, the CT state in AT is more coupled with L_b_ than L_a_, while in GC the converse is true. In both G and A, the coupling between L_b_ and L_a_ represents the most effective decay channel for L_b_ and, therefore, in both molecules an excitation to L_b_ first proceeds toward L_a_. However, while in GC the population arriving at L_a_ moves toward the CT, in AT this does not happen. Furthermore, the “total” electronic coupling of the CT state with the bright states of the purine (i.e., the sum of *E*_*ij*_^D^(0) values for L_a_-CT and L_b_-CT) is ∼20% larger for G than for A (see Table S11 in the SI).

**Figure 5:**
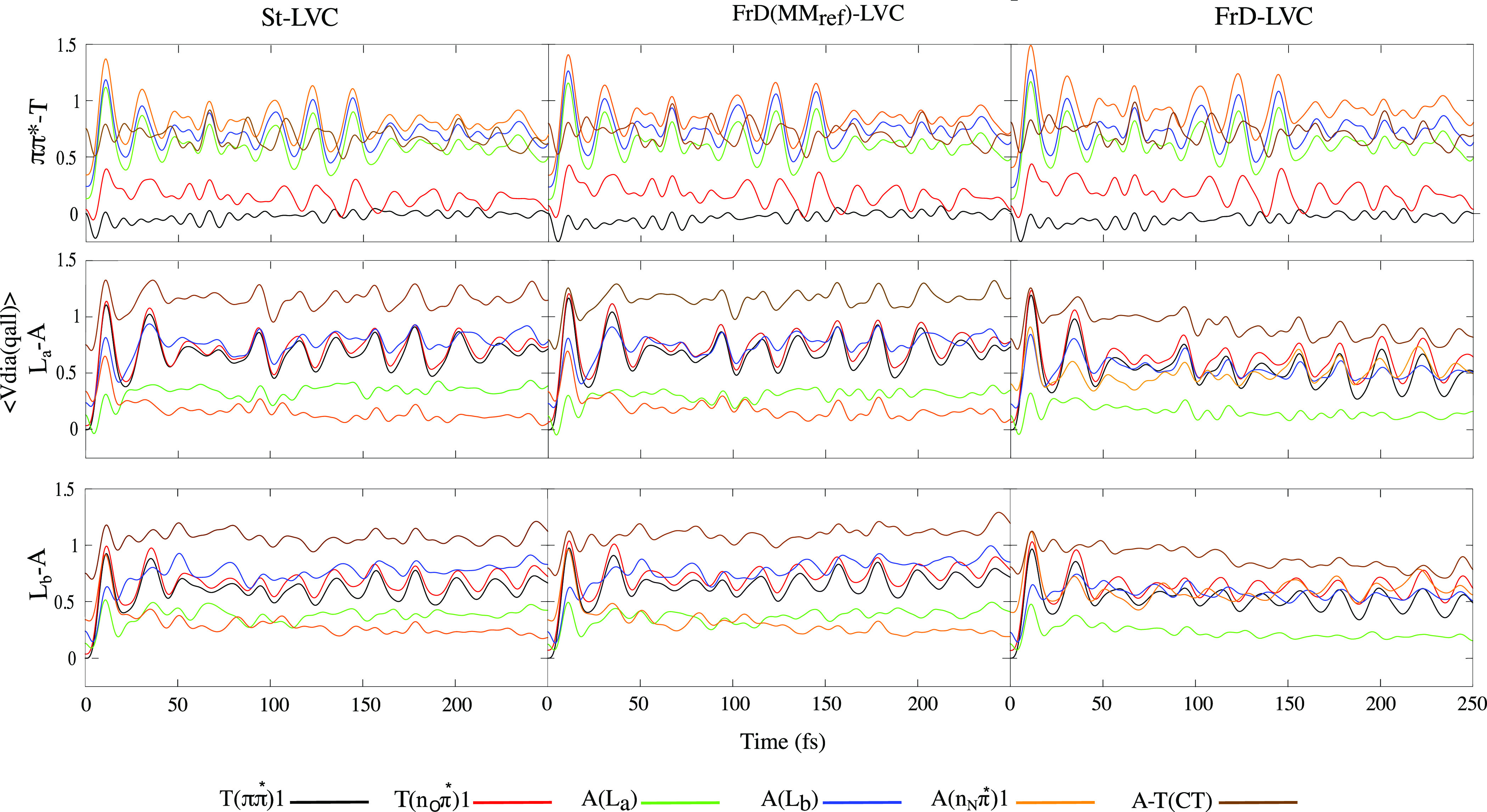
Expectation of diabatic PES for AT with initial excitation of T(ππ*1) (top), A(L_a_) (middle), and A(L_b_) (bottom) obtained by St-LVC (left), FrD(MM_ref_)-LVC (middle), and FrD-LVC(right). For clarity, only the first six diabatic states are shown. The full figure is shown in Figure S9 in the SI.

An analysis of the expectation values of the diabatic PES as a function of time in Figure 5 for the St-LVC (left) FrD(MM_ref_)-LVC (center) and FrD-LVC (right) models provides information coherent with the dynamics of the electronic populations discussed above. For an excitation to T(ππ*1) results are quite similar for the three models. The PES of T(ππ*1) and T(n_O_π*1) remain close in energy. They are always significantly more stable than the other states, in agreement with the fact that the dynamics basically involves these two states. In further detail, after being almost degenerate in the first few fs, they separate slightly until 70–80 fs in agreement with a slowing down of the transfer in Figure 4. At later times they approach each other again and even become degenerate before and after 150 fs when, however, the electronic populations have already reached an almost stationary value.

After a photoexcitation to A(L_a_), according to FrD-LVC the A(L_a_) energy is always lower than A(n_N_π*1), and they separate to an even greater extent at *t* > 70 fs when the transient population on A(n_N_π*1) starts flowing back to A(L_a_). On the contrary, according to St-LVC and FrD(MM_ref_)-LVC, for most of the time at *t* > 40 fs the energy of A(n_N_π*1) is lower than A(L_a_), in line with the fact that the population of the dark state becomes prevailing.

Finally, for a photoexcitation to A(L_b_), after the first ∼10 fs where A(L_a_) and A(L_b_) are quite similar and the L_b_ population flows to L_a_, the behavior of the expectation values of the diabatic energies becomes comparable to what is observed for a photoexcitation to L_a_.

For both these initial excitations on A, the CT diabatic potential is immediately destabilized by ∼0.5 eV in all the models. For the St-LVC and FrD(MM_ref_) models it remains approximately at this value for the duration of the dynamics. While for the FrD-LVC model it stabilizes to reach a value similar to that at the start, although it is still the highest energy state of the ones shown, ∼0.5 eV greater than A(L_a_).

#### Convergence of the Dynamics with the Number of Diabatic States: Photoexcitation to A(L_a_)

The 12-state models discussed in the previous subsection predict that, ∼50 fs after an initial excitation on A, the population of A(n_N_π*1) is much larger according to St-LVC and FrD(MM_ref_)-LVC than to FrD-LVC. This difference motivated a more in-depth methodological analysis of the long-time limit of the population of A(n_N_π*1), and to this end we performed calculations for an initial excitation to A(L_a_) on models with an increasing number of diabatic states.

**Figure 6:**
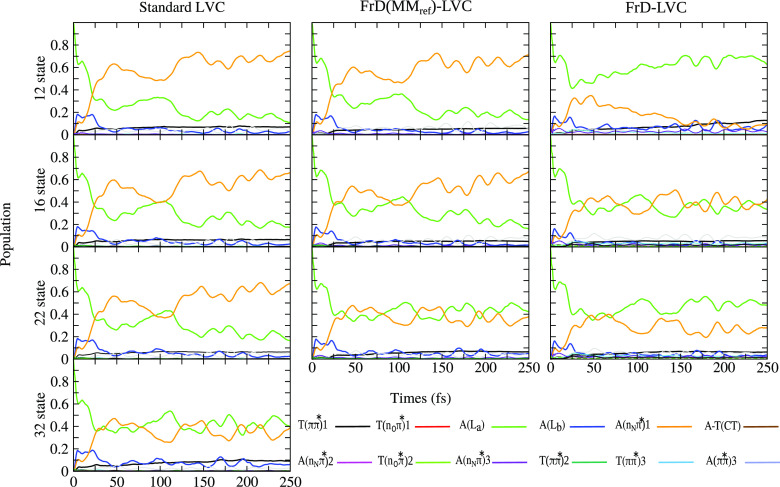
Diabatic-state populations for AT with initial excitation of A(L_*a*_) obtained with the standard (left) FrD(MM_ref_)-LVC (middle), and FrD-LVC (right) Hamiltonians including different numbers of diabatic states.

It should be realized that, when the number of states increases, establishing a one-to-one correspondence among the diabatic states included in the different models becomes more and more challenging. This was still possible up to 22 states (although with some caveats; see the SI), and in Figure 6 we compare the predictions of models including 12, 16, and 22 diabatic states, whose characters and adiabatic energies at the FC position are shown in Tables S13 and S19 in the SI, respectively. Increasing further the number of states, the one-to-one correspondence of the different models is lost and therefore we focus on the St-LVC model only, presenting a benchmark calculation including the lowest 32 states (up to an excitation of 8.3 eV). Further details may be found in the SI, section S3.5.

Figure 6 shows that, moving from 12 to 16 states, the FrD-LVC results become more similar to those of the other two models, predicting a partial, but not transient, population of A(n_N_π*1). Apart from this case, according to all LVC models, by increasing the number of diabatic states, A(L_a_) gains population and A(n_N_π*1) loses population. Moreover, independently of the number of states considered in the model, FrD-LVC tends to predict a smaller population of A(n_N_π*1) with respect to the other two diabatizations, due to the destabilization of its minimum (see the section “Excited-State Minima”). The SI shows similar trends for an initial excitation to A(L_b_) (Figure S10). In particular, also in this case the population of A(n_N_π*1) moderately decreases at the increase of the number of states. On the contrary, for an initial excitation to T(ππ*1), the population of the dark nπ* state, T(n_O_π*), increases with the number of diabatic states (Figure S11).

Focusing back on the results in Figure 6, according to the computations with the largest number of states (22, bottom panels), the populations of A(L_a_) and A(n_N_π*1) remain similar (∼40%) for all three models up to ∼100–120 fs. At longer times, according to FrD(MM_ref_)-LVC, A(L_a_) and A(n_N_π*1) populations do not show a further remarkable time evolution, whereas the population of A(n_N_π*1) decreases to 0.3 for FrD-LVC and increases up to 0.65 according to St-LVC. Considering the St-LVC model, the benchmark 32-state calculation still deviates from the 22-state one for *t* > 120 fs, but interestingly its predictions become much more similar to those of the two FrD approaches with 22 states.

In summary, Figure 6 documents the challenge to get fully converged results beyond 100 fs, when comparing models obtained with different diabatizations, and also calculations with the same diabatization scheme but different numbers of states. It should be noted that for the real photophysics of AT system, predictions of LVC models beyond the 100 fs time scale becomes less relevant, since in this time scale the neglected monomer-like decays to the ground state of both A and T are operative and compete with the decay channels investigated here.^3^ Notwithstanding this, these results are still interesting from the modellistic point of view. The different dynamics predicted by the three models could simply depend, at least in part, on the initial definition of the states. In fact, as discussed in previous sections, the states labeled A(L_a_) in the three models are similar but not identical. As a matter of fact, in Figure 3 we already showed that this leads to different contributions to the absorption spectrum from A(L_a_) and A(L_b_), although their sum is always very similar.

On the other side, it is really remarkable, and somewhat surprising, that a dynamics initiated on L_a_ can be altered by states lying almost 3 eV above, as shown generally in the changes from the 12- to 32-state calculations with the St-LVC models. The capability of high-lying states to affect dynamics initiated on L_a_ or L_b_ is confirmed even in the isolated A basis by the calculation with LVC model in Figure S12 in the SI, where we show that their presence reduces the long-time limit population of the A(n_N_π*) state from ∼90% to ∼80%. This finding raises some methodological concerns regarding LVC models in general, which we further discuss in the following conclusions section.

## Conclusions

In this contribution we have adopted LVC model Hamiltonians in combination with ML-MCTDH wavepacket propagations to investigate the ultrafast quantum dynamics of the AT base pair after a photoexcitation to the lowest bright states on thymine and adenine. We focused on the competition between the intermolecular excited-state processes, including CT states and excitation energy transfer, and the intramolecular decays from the bright states to dark nπ* states. We did not account for the direct monomer-like decays to the ground state which take place at conical intersections occurring at molecular structures too distorted to be described with LVC models. Furthermore, the adoption of LVC Hamiltonians limits the reliable prediction of the photophysics to the ultrafast (100 fs) time scale, where the wavepacket mainly explores planar configurations. However, the information gained on which states are populated in these times can give indications on possible avenues for subsequent decay processes. Moreover, with ever increasing resolution in pump–probe and 2D spectroscopies, unravelling these ultrafast population transfers is important to aid experimental assignment.

The main outcome of our study is to show that the intramolecular decays to the nπ* states are dominant over the intermolecular processes. These intramolecular pathways are the same that are operative in the <100 fs dynamics of isolated A and T in the gas phase, although their yield is reduced in the AT pair, due to the fact that hydrogen bonding destabilizes the nπ* states. The population of CT states is negligible, at least in the fs time regime, in marked contrast with what happens in the GC base pair,^49^ suggesting that the ultrafast PCET decay mechanism is not operative in AT. It will be interesting in the future to revisit this conclusion by adopting more refined model Hamiltonians, accounting for instance for the anharmonicity of H-bond vibrations that clearly change remarkably in a CT state with respect to what is assumed by the LVC model. In this respect, it is noteworthy that a TD-DFT optimization indicates that LVC models slightly overestimate the CT minimum energy by ∼0.1 eV (St-LVC value). The excitation energy transfer from bright states on A to those on T is also predicted to be a minor pathway (<10%), and the transfer from T to A is predicted to be even smaller (<5%). It is also conceivable that, if the nπ* states live long enough in the time scale from pico- to nanoseconds, alternative channels toward the population of the CT state could be open, making the PCET mechanism feasible. Due to the likelihood of large amplitude motions occurring on this time scale, this possibility cannot be investigated with a LVC model Hamiltonian.

Other potential interesting future investigations involving AT could concern the dynamics exhibited by tautomers other than the WC arrangement, in order to have a direct connection to gas phase experiments,^77,78^ where the WC arrangement is not predicted to be the most stable.^11^ Note, however, that the most stable gas phase structure is not possible for the 9-methyladenine we use as a molecular model in this work, as it involves a hydrogen bond on N9–H. Comparison to a Hoogsteen bonded pair could also be worthwhile, although this is much less prevalent in DNA than the WC arrangement. Finally, the dynamics involving the fluorescent analogue of A, 2-aminopurine, could also be worthwhile to investigate and compare, since it can be inserted into DNA without disrupting the helical structure, is often used as a molecular probe, and its dynamics in WC and Hoogsteen arrangements with T have recently been investigated experimentally.^10^

From the methodological point of view, our results further confirm the effectiveness of our protocols to parametrize St-LVC and FrD-LVC Hamiltonians. For MC assemblies, they make it possible to define diabatic states either from states of the full system computed at a given structure (e.g., the ground-state minimum), or rigorously localized states built for the fragments. The former ones are eigenstates of the electronic Hamiltonian (at least at that geometry) but may be partially delocalized; the latter ones are fully localized but are not in principle eigenstates of the MC Hamiltonian. Which of the two strategies provides the most realistic description of the dynamics will depend on the system and the issue under investigation. It is fundamental, for instance, to explicitly address the problem of the excitation process, i.e., the preparation of the initial state. An excitation with a narrow laser pulse in the frequency domain (long in the time domain) probably excites a single, in principle delocalized, adiabatic electronic state and is therefore better described by a standard LVC model. On the other side, in the limit of an excitation with a very broad pulse in the frequency, the so-called doorway state is excited, and it may be better described in a fragment picture. More elaborated pulses, like those produced with pulse shapers can prepare even more complex initial states so that in general a complete analysis of these issues can only be obtained by explicitly including the interaction with the laser pump field in the Hamiltonian. It is also possible that depending on the particular scientific questions, the answer can be more straightforwardly found within a fragment or a delocalized (standard) picture.

In this contribution we also introduced a variation on the FrD-LVC approach, which we named FrD(MM_ref_)-LVC. It allows us to keep our chemically intuitive description of diabatic states for MC systems in terms of individual chromophores, while at the same time accounting for the electrostatic effects of the surrounding chromophores on the local excitations and orbitals in a MM fashion. In this way, it is possible to define “in situ” monomer-like fragments. In general, it should be advantageous to describe the surroundings in such a way when defining the diabatic states, as there is only a limited additional cost in computing the MM charges. When the chromophores are far away from each other, this will, however, only have a small effect. For the specific AT system, where the adiabatic states are quite well localized, this strategy allowed for a more similar description of excited-state minima and population dynamics to the standard LVC approach. In systems where this is not the case, such as duplex DNA with stacking as well as hydrogen bonding interactions, the FrD(MM_ref_)-LVC approach can be a useful tool in understanding the dynamics in terms of individual sites. Furthermore, the potentialities of using a QM/MM approach in combination with the diabatic electronic states can allow the consideration of the DNA backbone, ions, and solvent.

The effectiveness of the parametrization of the LVC models, coupled with the impressive capabilities of the ML-MCTDH methodology, make it nowadays feasible to investigate the dynamics of systems with several coupled electronic states with different nature, like in AT, considering all the vibrational degrees of freedom. On the other hand, this possibility leads us to face with several “unexplored” features, perhaps not yet emerged due to previous computational limitations. For example, we have shown that increasing the number of diabatic states included in the model can alter the time evolution of the electronic populations of lower lying states. Two possible conclusions can be drawn from this: (i) that the manifold of adiabatic states at energies of interest for the WP could actually get contributions also from diabatic states quite high in energy or (ii) that this phenomenon may be pathological result of the linear approximation of the couplings (which therefore progressively increase with the displacement from the FC position). These factors should be considered in tailored future studies, in particular to determine how robust the determination of couplings among states with large energy gaps is with respect to the adopted level of electronic structure theory. Furthermore, our results suggest that when a few states have minima at very similar energies, small energy shifts can significantly affect the population dynamics. It will be interesting to investigate, in the future, if this prediction is confirmed also with more accurate potentials than the LVC model. In summary, the approaches presented here provide efficient explorations of the early time excited state dynamics in complex MC systems, allowing us to individuate the key physicochemical effects and the most important electronic states and vibrational modes. They can also represent a first step for designing more accurate investigations targeting longer time processes and adopting more refined potentials.

## Supporting Information Available

Details on ML-MCTDH trees and convergence checks, more analysis on the nature of the states in the ground and excited state minima, tables reporting diabatic energies, couplings, and LVC adiabatic energies and eigenvectors, analysis of vibrational dynamics, additional data on the study of the convergence of the results with the number of diabatic states, and QD of the isolated bases (PDF)
