# Supplementary material for: The Ultrafast Quantum Dynamics of Photoexcited Adenine–Thymine Basepair Investigated with a Fragment-based Diabatization and a Linear Vibronic Coupling Model
Source: J Phys Chem A. 2021 Oct 5;125(40):8912–24. doi: 10.1021/acs.jpca.1c08132 (PMC9281421; doi:10.1021/acs.jpca.1c08132)
Supplement: Supplementary file 1 — jp1c08132_si_001.pdf [file jp1c08132_si_001.pdf]

Supporting Information: The Ultrafast Quantum Dynamics of Photoexcited Adenine-Thymine Basepair Investigated with a Fragment-based Diabatization and a Linear Vibronic Coupling Model

Martha Yaghoubi Jouybari,[†] James A. Green,[‡] Roberto Improta,[*,‡] and Fabrizio Santoro[*,†]

†*Consiglio Nazionale delle Ricerche, Istituto di Chimica dei Composti Organo Metallici (ICCOM-CNR), SS di Pisa, Area della Ricerca, via G. Moruzzi 1, I-56124 Pisa, Italy*

‡*Consiglio Nazionale delle Ricerche, Istituto di Biostrutture e Bioimmagini (IBB-CNR), via Mezzocannone 16, I-80136 Napoli, Italy*

E-mail: robimp@unina.it; fabrizio.santoro@pi.iccom.cnr.it

Contents

S1 Additional Computational Details **S-3**

S1.1 On the different references states for diabatization S-3

S1.2 Absorption Spectra S-4

S2 Convergence checks **S-5**

S2.1 Convergence test of the ML-MCTDH calculations S-5

S3 Further results **S-7**

S3.1 FC point S-7

S3.1.1 L_a and L_b states of Adenine S-11

S3.2 Adiabatic and Diabatic Minima S-15

S3.3 Dynamics S-21

S3.3.1 Reference results for the dynamics of 1methyl-Thymine and 9methyl-AdenineS-21

S3.3.2 Population of CT: AT vs GC S-22

S3.4 Time-dependence of diabatic potentials S-23

S3.5 Study of the convergence of the electronic population dynamics with the number of diabatic states S-24

S3.5.1 FrD(MM_{ref})-LVC calculations S-25

S3.5.2 FrD-LVC calculations S-31

S3.5.3 Photoexcitation to A(L_b) and T($\pi\pi^*$) S-35

S3.5.4 Effect of the increase of the number of diabatic states on isolated AdenineS-37

References **S-38**

S1 Additional Computational Details

S1.1 On the different references states for diabatization

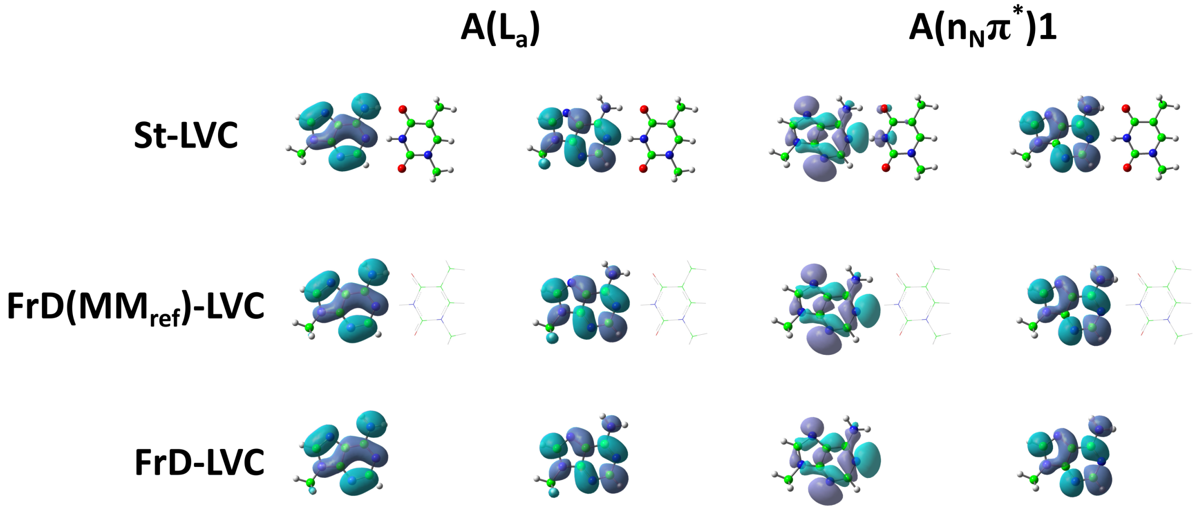

Figure S1: A pictorial representation of the three different choices of the references states adopted for diabatization in St-LVC, FrD(MM_{ref})-LVC and FrD-LVC parameterization for two states localized on Adenine. In FrD-LVC, these states are computed for the isolated Adenine in the geometry it has in the AT ground state minimum. FrD(MM_{ref})-LVC is similar to FrD but the reference states are computed accounting for the effect of the thymine at MM level (for single-point calculations this is equivalent to describe thymine as a set of charges, we chose RESP protocol). In St-LVC we adopt as reference states the adiabatic states of the AT base pair computed at the ground state geometry. They are denoted with the same labels of the fragment states they resemble most. Of course this is possible in AT where the adiabatic states are already well-localized, but may not be possible in general. In fact, in other systems (e.g. stacked dimers of nucleobases) such states might be strongly delocalized, making impossible establishing a one-to-one correspondence with fragment states.

S1.2 Absorption Spectra

For the st-LVC model, the transition dipoles of the diabatic states $\boldsymbol{\mu}[\boldsymbol{d}]$ are simply those of the adiabatic states of the MC. For the FrD-LVC approaches, the transition dipole moments of the diabatic states at the reference geometry may be obtained by applying the transformation matrix $\boldsymbol{D}$, defined in Eq. 5 in the main text, to the diagonal matrix of adiabatic transition dipole moments of the MC at reference geometry $\boldsymbol{\mu}[\boldsymbol{a}^{\mathsf{MC}}]$

$$\boldsymbol{\mu}[\boldsymbol{d}] = \boldsymbol{D}^T \boldsymbol{\mu}[\boldsymbol{a}^{\mathsf{MC}}]\boldsymbol{D}. \tag{S1}$$

Then, for both st-LVC and FrD-LVC approaches, the absorption spectra $\epsilon(\omega)$ at zero Kelvin, can be expressed in a TD framework as:

$$\begin{aligned} \epsilon(\omega) &= \frac{2\pi\omega N_{\mathsf{A}}}{3000 \times \ln 10 \times \hbar c_0 (4\pi\epsilon_0)} \sum_{ji} \int_{-\infty}^{\infty} dt e^{i\omega t - \Gamma t^2} \langle \mathbf{0}; d_j | \boldsymbol{\mu}^{\mathsf{d}}_{gj} e^{-i\hat{H}t/\hbar} \boldsymbol{\mu}^{\mathsf{d}}_{ig} | d_i; \mathbf{0} \rangle \\ &= \sum_i \epsilon_{ii}(\omega) + \sum_{i,j\neq i} \epsilon_{ij}(\omega) = \epsilon^{\mathsf{auto}}(\omega) + \epsilon^{\mathsf{cross}}(\omega) \end{aligned} \tag{S2}$$

where N_{A} is Avogadro's number, c_0 is the speed of light in vacuo, ϵ_0 is the vacuum permittivity and we introduced a quadratic damping ruled by a parameter Γ, corresponding to a Gaussian broadening in the frequency domain. We represent the diabatic transition dipole moments with the shorthand $\boldsymbol{\mu}^{\mathsf{d}}_{gj}$ for the transition from ground state g to diabatic state d_j in the above. The diabatic transition dipole moment is considered independent of the nuclear coordinates (Condon approximation), and the ground-vibrational state of the ground electronic state is represented by $\mathbf{0}$ and its energy is set to zero.

The auto (ϵ^{auto}) and cross ($\epsilon^{\mathsf{cross}}$) correlation functions are obtained by numerical propagation in time under the effect of the LVC Hamiltonian of the doorway states $|d_j; \mathbf{0}\rangle$ obtained by a vertical excitation of the vibrational state $\mathbf{0}$ to the bright diabatic states (i.e. for which $\boldsymbol{\mu}_{ig}$ is non vanishing). Then, the vibronic absorption spectrum is obtained by Fourier transform of the sum

of the correlations functions weighted by the scalar products of the diabatic transition dipoles (as reported in Equation S2). Cross-correlations functions usually have a very small effect and have been neglected.[S1,S2]

S2 Convergence checks

S2.1 Convergence test of the ML-MCTDH calculations

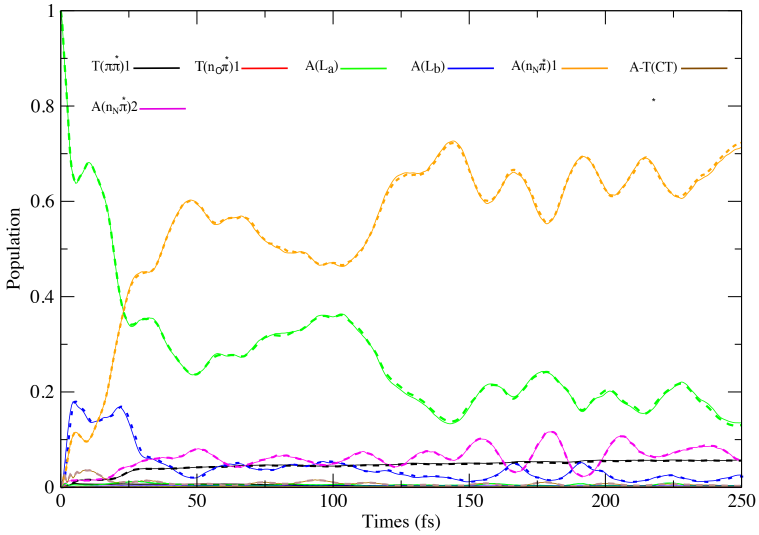

Figure S2: Convergence test for ML-MCTDH propagations with respect to the number of single particle functions (SPFs). We considered the nonadiabatic dynamics of electronic populations of AT in gas phase predicted with FrD(MM_{ref})-LVC Hamiltonians parameterized at the FC point with CAM-B3LYP calculations. The time evolution adopted with the settings used in most of the calculations ("standard", solid line) is compared with what obtained decreasing both the number of SPFs (dash line). A graphical representation of the multilayer (ML) trees is reported in Figure S3. Similar tests were done modifying the dimension of the primitive basis set, showing also in this case a good convergence. Data not reported

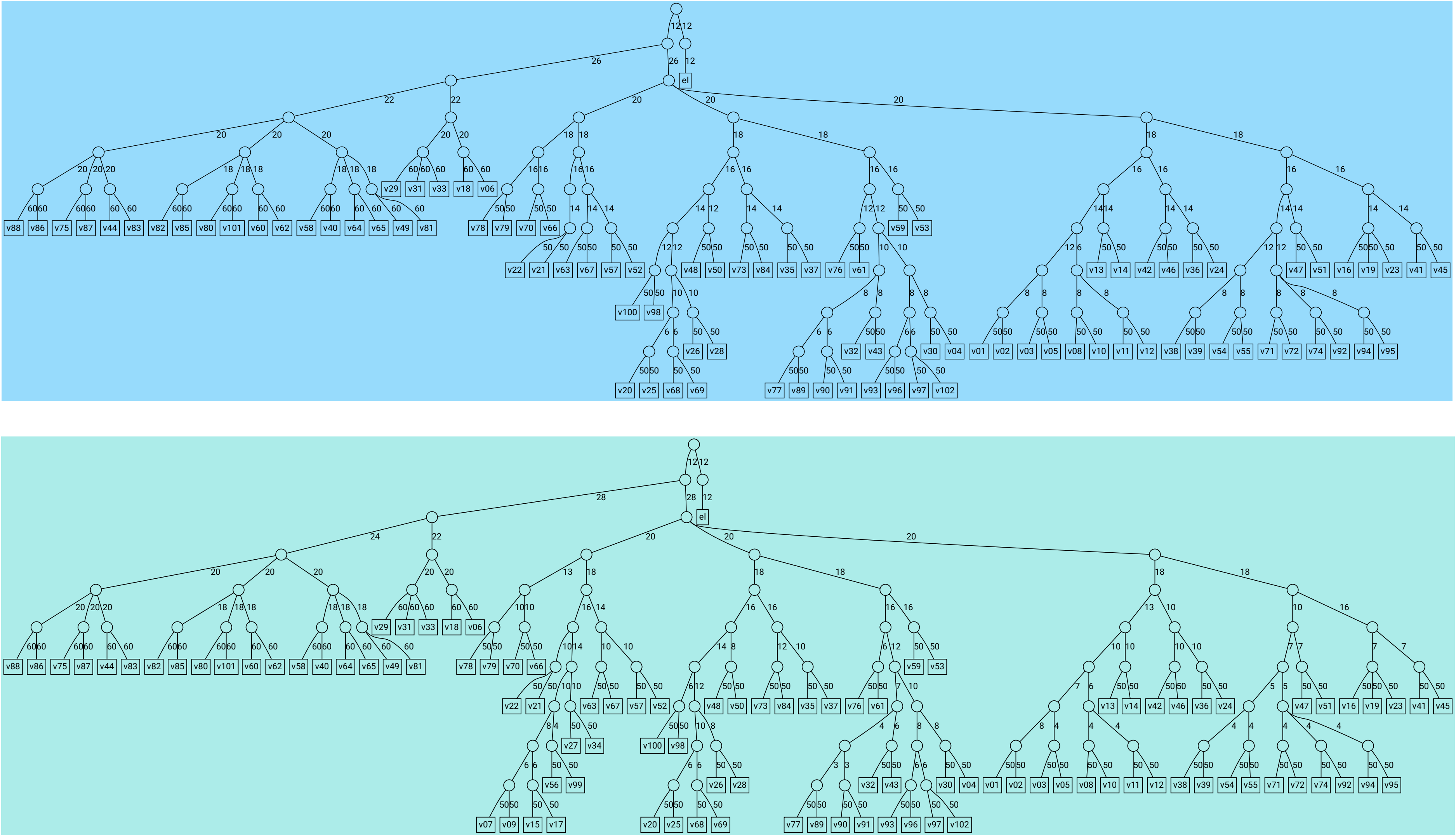

Figure S3: Graphical representation of a typical ML-MCTDH tree adopted in the computations (top) and with smaller basis set and number of SPFs adopted to check the convergence of the calculations made with standard settings (bottom)

S3 Further results

S3.1 FC point

Table S1: Symmetry (Sym), electronic characters, energies ($E_m^{\text{A,DFT}}$, in eV, with respect to S_0 in the FC point), weight of the predominant diabatic state in adiabatic state for both FrD(MM$_{\text{ref}}$)-LVC and FrD-LVC (W_{RESP}, W_{isolated}), and oscillator strengths δ_{OPA}, computed for AT at the FC point. CAM-B3LYP/6-31G(d) calculations. This is an extended version of the Table 1 in the main text, showing also the predominant transition coefficients in terms of the Kohn-Sham orbitals. Orbital numbering shown in Figure S4.

State	Sym	Character	$E_m^{\text{A,DFT}}$	W_{RESP}	W_{isolated}	δ_{OPA}	Trans.	Coeff.
S_1	A'	T($\pi\pi^*$1)	5.32	0.96	0.95	0.204	75 → 77	0.674
S_2	A"	T($n_O\pi^*$1)	5.36	0.99	0.99	0.000	72 → 77	0.569
							74 → 77	0.242
S_3	A'	A(L_a)	5.45	0.98	0.94	0.126	76 → 79	0.489
							76 → 78	0.429
S_4	A'	A(L_b)	5.56	0.95	0.93	0.203	76 → 78	0.512
							76 → 79	-0.367
S_5	A"	A($n_N\pi^*$1)	5.66	0.99	0.91	0.000	74 → 78	0.649
S_6	A'	A→T(CT)	6.08	0.97	0.97	0.003	76 → 77	0.676
S_7	A"	A($n_N\pi^*$2)	6.14	0.99	0.96	0.000	74 → 79	0.656
S_8	A"	T($n_O\pi^*$2)	6.44	0.99	0.99	0.000	72 → 80	0.445
							69 → 80	-0.367
S_9	A"	A($n_N\pi^*$2)	6.61	0.99	0.91	0.003	69 → 78	0.484
							68 → 78	0.355
S_{10}	A'	T($\pi\pi^*$2)	6.63	0.85	0.85	0.017	71 → 77	0.597
							73 → 78	0.224
S_{11}	A'	T($\pi\pi^*$3)	6.73	0.84	0.83	0.296	75 → 80	0.639
S_{12}	A'	A($\pi\pi^*$3)	6.81	0.71	0.71	0.268	73 → 78	0.474
							75 → 80	-0.269
							76 → 79	0.258
							71 → 77	-0.249

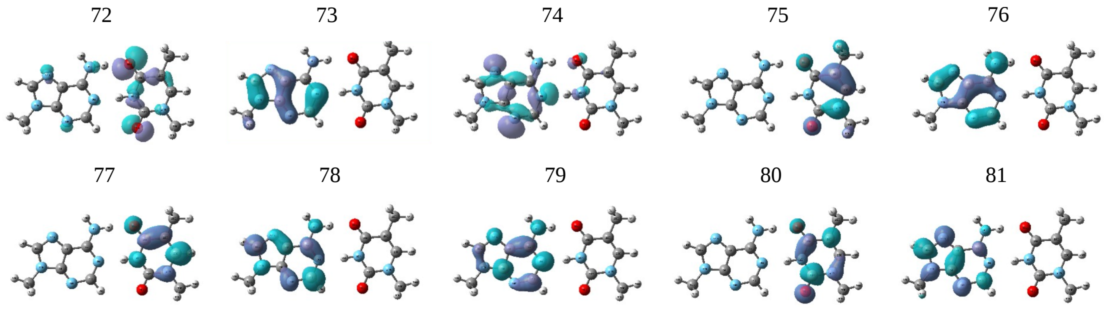

Figure S4: Schematic drawing of the main Kohn-Sham molecular orbitals of AT involved in the electronic transitions discussed in the present paper, computed at CAM-B3LYP/6-31G(d) level of theory with isovalue 0.04.

Table S2: Eigenvalues (eV) of A' adiabatic LVC states and corresponding normalised eigenvectors showing the contribution of the $\pi\pi^*$ diabatic states from the 12 state FrD(MM$_{\text{ref}}$)-LVC and FrD-LVC models of AT in C_{s} symmetry at the FC point. Parametrized by CAM-B3LYP with 6-31G(d) basis set.

	FrD(MM$_{\text{ref}}$)-LVC						
Adiabatic states →	S_1	S_3	S_4	S_6	S_{10}	S_{11}	S_{12}
Eigenval. (eV) →	5.326	5.457	5.567	6.136	6.644	6.729	6.813
Eigenvec. ↓							
T($\pi\pi^*$1)	0.982	-0.081	-0.166	0.010	-0.013	-0.014	0.032
A(L$_{\text{a}}$)	0.096	0.990	0.085	-0.026	-0.013	-0.035	-0.033
A(L$_{\text{b}}$)	0.156	-0.095	0.975	0.118	0.046	-0.008	0.009
A→T(CT)	-0.027	0.035	-0.106	0.987	-0.115	-0.021	-0.015
T($\pi\pi^*$2)	0.013	0.002	-0.054	0.095	0.922	-0.106	-0.356
T($\pi\pi^*$3)	0.028	0.016	0.009	0.009	-0.048	0.918	-0.393
A($\pi\pi^*$3)	-0.017	0.051	-0.021	0.059	0.364	0.380	0.847

	FrD-LVC						
Adiabatic states →	S_1	S_3	S_4	S_6	S_9	S_{11}	S_{12}
Eigenval. (eV) →	5.327	5.459	5.571	6.157	6.646	6.731	6.817
Eigenvec. ↓							
T($\pi\pi^*$1)	0.979	-0.088	-0.158	0.006	-0.050	-0.047	0.064
A(L$_{\text{a}}$)	0.115	0.974	0.163	-0.019	-0.028	-0.057	-0.080
A(L$_{\text{b}}$)	0.140	-0.171	0.966	0.117	0.056	0.008	0.041
A→T(CT)	-0.025	0.033	-0.102	0.986	-0.119	-0.025	-0.016
T($\pi\pi^*$2)	0.051	-0.004	-0.059	0.098	0.925	-0.143	-0.329
T($\pi\pi^*$3)	0.071	0.012	-0.000	0.017	-0.008	0.912	-0.403
A($\pi\pi^*$3)	-0.016	0.112	-0.044	0.057	0.352	0.376	0.847

Table S3: Eigenvalues (eV) of A" adiabatic LVC states and corresponding normalised eigenvectors showing the contribution of the nπ^* diabatic states from the 12 state FrD(MM$_{ref}$)-LVC and FrD-LVC models of AT in C_s symmetry at the FC point. Parametrized by CAM-B3LYP with 6-31G(d) basis set.

	FrD(MM$_{ref}$)-LVC				
Adiabatic states →	S_2	S_5	S_7	S_8	S_9
Eigenval. (eV) →	5.406	5.672	6.152	6.444	6.626
Eigenvec. ↓					
T($n_O\pi^*1$)	0.999	-0.007	-0.003	-0.002	-0.001
A($n_N\pi^*1$)	0.007	0.997	-0.073	0.002	0.037
A($n_N\pi^*2$)	0.004	0.075	0.996	-0.012	-0.045
T($n_O\pi^*2$)	0.002	-0.002	0.013	0.999	0.008
A($n_N\pi^*3$)	0.001	-0.033	0.047	-0.009	0.998

	FrD-LVC				
Adiabatic states →	S_2	S_5	S_7	S_8	S_{10}
Eigenval. (eV) →	5.415	5.679	6.206	6.450	6.658
Eigenvec. ↓					
T($n_O\pi^*1$)	0.999	-0.008	-0.004	0.002	-0.001
A($n_N\pi^*1$)	0.007	0.956	-0.122	-0.002	0.268
A($n_N\pi^*2$)	0.005	0.157	0.981	0.016	-0.116
T($n_O\pi^*2$)	-0.002	0.001	-0.017	0.999	-0.006
A($n_N\pi^*3$)	-0.000	-0.248	0.152	0.009	0.957

Table S4: Diabatic energies ($E^{\mathrm{D}}_{ii}(0)$) and electronic couplings ($E^{\mathrm{D}}_{ij}(0)$) of A' diabatic states of AT in C_s symmetry at ground state equilibrium geometry from the FrD(MM$_{ref}$)-LVC 12 model (eV). Results for the FrD-LVC model in parentheses.

STATE	T ($\pi\pi^*1$)	A (L_a)	A (L_b)	A→T (CT)	T ($\pi\pi^*2$)	T ($\pi\pi^*3$)	A ($\pi\pi^*3$)
T($\pi\pi^*1$)	5.336(5.347)						
A(L_a)	-0.015(-0.020)	5.460(5.475)					
A(L_b)	-0.037(-0.035)	0.004(0.007)	5.570(5.577)				
A→T(CT)	0.014(0.016)	-0.015(-0.007)	0.062(0.061)	6.135(6.157)			
T($\pi\pi^*2$)	-0.028(-0.079)	0.004(0.013)	0.048(0.043)	-0.052(-0.051)	6.658(6.655)		
T($\pi\pi^*3$)	-0.036(-0.098)	-0.023(-0.023)	-0.015(-0.014)	-0.004(-0.008)	0.015(0.006)	6.740(6.737)	
A($\pi\pi^*3$)	0.027(0.033)	-0.061(-0.132)	0.029(0.074)	-0.037(-0.040)	-0.058(-0.056)	-0.027(-0.030)	6.771(6.762)

Table S5: Diabatic energies ($E^{\mathsf{D}}_{ii}(0)$) and electronic couplings ($E^{\mathsf{D}}_{ij}(0)$) of A" diabatic states of AT in C_{s} symmetry at ground state equilibrium geometry from the FrD(MM$_{\mathsf{ref}}$)-LVC 12 model (eV). Results for the FrD-LVC model in parentheses.

STATE	T<br>($n_O\pi^*1$)	A<br>($n_N\pi^*1$)	A<br>($n_N\pi^*2$)	T<br>($n_O\pi^*2$)	A<br>($n_N\pi^*3$)
T($n_O\pi^*1$)	5.406(5.415)				
A($n_N\pi^*1$)	-0.002(-0.002)	5.675(5.757)			
A($n_N\pi^*2$)	-0.003(-0.003)	-0.037(-0.093)	6.150(6.199)		
T($n_O\pi^*2$)	-0.003(0.001)	0.002(-0.002)	-0.004(0.004)	6.444(6.450)	
A($n_N\pi^*3$)	-0.001(-0.001)	0.033(0.241)	-0.020(-0.029)	0.001(-0.000)	6.624(6.587)

S3.1.1 L_a and L_b states of Adenine

Inspection of Table S1 and Figure 2 in the main text show that S_3 and S_4 are clearly associated to two bright excited states localized on A, the latter being slightly more intense of the former. In the isolated Adenine in its ground state minimum, L_a is expected to be the stronger absorbing state with a dominant H→L character, and L_b the weaker absorbing one with a dominant H→L+1 character.[S3] The relative position of these two bands is still the subject of a very lively debate, since different methods give different predictions.[S3,S4] For isolated A, in the geometry that it appears in the AT minimum (i.e. the geometry used for the reference states in the FrD-LVC approach), Figure S5 shows that at the selected level of theory, the two main configurations H→L, H→L+1 are strongly mixed in the two bright adiabatic states. However, the most stable and most absorbing state is the one with larger H→L component, which we therefore we label as L_a.

In the base pair, this picture appears to be switched, as the lower energy state (S_3) is less absorbing and with a greater H→L+1 component, corresponding to the transition between KS orbitals of 76→79. The higher energy state (S_4), is more absorbing, and has a larger component from the KS orbitals 76→78 corresponding to H→L in isolated A.

For A in the field of the RESP charges of T (i.e. the procedure used for the reference states in the FrD(MM_{ref}) approach) things are again different. As in the base pair, the more stable state (S_1) is the weaker, but in this case its H→L coefficient is larger than H→L+1, whereas S_2 is more intense but with a larger component H→L+1.

The overlap matrices computed in the diabatization procedure provide a way to quantitatively asses the correspondence between the A-like states in AT and those of the isolated monomer (see Table S1) The projections of the L_a and L_b states in the FrD-LVC approaches on S_3 and S_4 of the base pair are respectively 94% and 93%. For FrD(MM_{ref})-LVC reference states these projections become 98% and 95%. Whereas the above projections are large and therefore assignments are robust, it is noteworthy that the overlap matrix (see Table S6) also indicates that the TD-DFT states of the base pair (and therefore also the diabatic states of the St-LVC model) actually have

some small components from the other state of Adenine (L_b or L_a) and even from the lowest $\pi\pi^*$ state of T.

Table S6: Overlaps between the TD-DFT adiabatic states of the base pair computed at the FC position and the local diabatic states corresponding to the first bright state of Thymine, T($\pi\pi^*$), and the first two bright states of Adenine, A(L_a) and A(L_b), as defined with Standard, FrD or FrD(MM_{ref}) LVC approaches.

	Standard			FrD(MM_{ref})			FrD		
Diabatic states→	T($\pi\pi^*$)	A(L_a)	A(L_b)	T($\pi\pi^*$)	A(L_a)	A(L_b)	T($\pi\pi^*$)	A(L_a)	A(L_b)
Adiabatic ↓									
S1 [T($\pi\pi^*$)]	1.000	0.000	0.000	0.992	0.092	0.162	0.987	0.108	0.151
S3 [A(L_a)]	0.000	1.000	0.000	-0.075	0.995	-0.094	-0.076	0.976	0.183
S4 [A(L_b)]	0.000	0.000	1.000	-0.175	0.091	0.979	-0.172	0.184	0.962

Base Pair			Single Base Adenine-RESP			Single Base Adenine		
State 3<br>La<br><br>5.4537 eV<br><br>f=0.126	76-78 0.42<br>76-79 0.48<br>73-78 -0.23	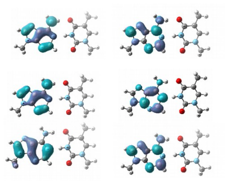	State 1<br>La<br><br>5.4994 eV<br><br>f=0.126	39-40 0.47<br>39-41 0.44<br>37-40 -0.24<br>37-41 0.11	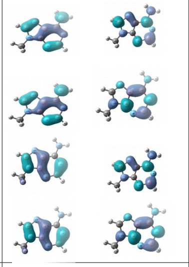	State 1<br>La<br><br>5.5107 eV<br><br>f=0.170	39-40 0.55<br>39-41 0.35<br>37-40 -0.20<br>37-41 0.13	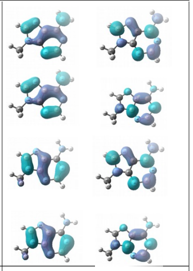
State 4<br>Lb<br><br>5.5618 eV<br><br>f=0.203	73-78 0.2<br>73-79 0.1<br>75-77 -0.1<br>76-77 -0.13<br>76-78 0.51<br>76-79 -0.36	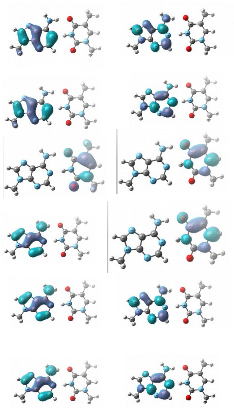	State 2<br>Lb<br><br>5.5949 eV<br><br>f=0.168	39-40 0.49<br>39-41 -0.40<br>37-40 0.26<br>37-41 0.1	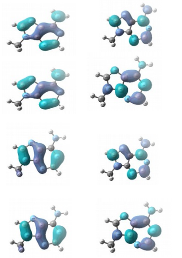	State 2<br>Lb<br><br>5.600 eV<br><br>f=0.112	39-40 -0.40<br>39-41 0.46<br>37-40 -0.32	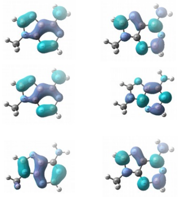
State 5<br>$n_N$pi*<br><br>5.6629 eV	74-78 0.64<br>74-79 0.15<br>74-81 -0.12<br>72-78 -0.10	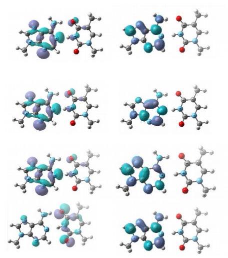	State 3<br>$n_N$pi*<br><br>5.6427 eV	38-40 0.66<br>38-41 0.13<br>38-42 -0.13	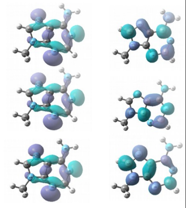	State 3<br>$n_N$pi*<br><br>5.4492 eV	38-40 0.67<br>38-42 -0.15	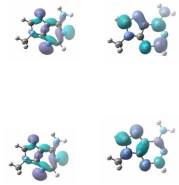

Figure S5: Electronic character and main orbital transitions corresponding to states we label as L_a, L_b and $n_N\pi^*$ states in the adiabatic set (TD-DFT on the baseair)(left) and diabatic basis set (TD-DFT on single base Adenine) in the field of RESP charges(middle) and without RESP charge(right).

Table S7: Norm of coupling vector $\sqrt{\boldsymbol{\lambda}_{ij} \cdot \boldsymbol{\lambda}_{ij}}$ for 12 diabatic state LVC models of AT in C_s symmetry at ground state equilibrium geometry. Differences between the models are highlighted in bold.

STATE	1	2	3	4	5	6	7	8	9	10	11	12
	St LVC											
1 T($\pi\pi^*$1)	0.333											
2 T($n_O\pi^*$1)	0.062	0.395										
3 A(L_a)	**0.048**	0.005	0.319									
4 A(L_b)	**0.076**	0.014	0.153	0.277								
5 A($n_N\pi^*$1)	0.017	0.008	0.104	0.068	0.419							
6 A→T(CT)	0.021	0.052	0.030	0.074	0.012	0.569						
7 A($n_N\pi^*$2)	0.012	0.013	0.060	0.081	0.225	0.006	0.362					
8 T($n_O\pi^*$2)	0.040	0.092	0.005	0.008	0.005	0.006	0.014	0.492				
9 A($n_N\pi^*$3)	0.022	0.008	0.073	0.109	0.216	0.018	0.116	0.008	0.315			
10 T($\pi\pi^*$2)	0.100	0.067	**0.066**	**0.095**	0.032	0.069	0.025	0.021	0.041	0.418		
11 T($\pi\pi^*$3)	0.167	0.033	**0.063**	**0.092**	0.027	0.017	0.031	0.071	0.032	0.049	0.354	
12 A($\pi\pi^*$3)	**0.096**	0.033	0.138	0.196	0.061	0.040	0.060	0.039	0.070	**0.188**	**0.175**	0.178
	FrD(MM_{ref})-LVC											
1 T($\pi\pi^*$1)	0.344											
2 T($n_O\pi^*$1)	0.059	0.422										
3 A(L_a)	**0.004**	0.003	0.318									
4 A(L_b)	**0.006**	0.008	0.151	0.279								
5 A($n_N\pi^*$1)	0.002	0.002	0.106	0.067	0.406							
6 A→T(CT)	0.006	0.044	0.007	0.012	0.001	0.605						
7 A($n_N\pi^*$2)	0.002	0.002	0.057	0.081	0.227	0.001	0.370					
8 T($n_O\pi^*$2)	0.040	0.098	0.002	0.002	0.002	0.004	0.002	0.494				
9 A($n_N\pi^*$3)	0.001	0.002	0.081	0.113	0.216	0.001	0.105	0.001	0.316			
10 T($\pi\pi^*$2)	0.111	0.076	**0.005**	**0.008**	0.005	0.011	0.009	0.026	0.006	0.502		
11 T($\pi\pi^*$3)	0.186	0.036	**0.003**	**0.004**	0.002	0.002	0.002	0.080	0.002	0.063	0.420	
12 A($\pi\pi^*$3)	**0.004**	0.006	0.184	0.241	0.076	0.007	0.072	0.004	0.085	**0.009**	**0.004**	0.211
	LVC-FrD											
1 T($\pi\pi^*$1)	0.346											
2 T($n_O\pi^*$1)	0.058	0.424										
3 A(L_a)	0.004	0.004	0.315									
4 A(L_b)	0.005	0.007	0.143	0.264								
5 A($n_N\pi^*$1)	0.002	0.003	0.098	0.061	0.405							
6 A→T(CT)	0.006	0.043	0.006	0.011	0.001	0.607						
7 A($n_N\pi^*$2)	0.002	0.003	0.044	0.067	0.240	0.002	0.356					
8 T($n_O\pi^*$2)	0.041	0.105	0.002	0.002	0.002	0.005	0.003	0.485				
9 A($n_N\pi^*$3)	0.002	0.002	0.102	0.127	0.205	0.001	0.069	0.001	0.311			
10 T($\pi\pi^*$2)	0.104	0.076	0.005	0.008	0.007	0.011	0.010	0.024	0.005	0.496		
11 T($\pi\pi^*$3)	0.189	0.039	0.003	0.004	0.003	0.002	0.003	0.081	0.002	0.108	0.412	
12 A($\pi\pi^*$3)	0.004	0.006	0.206	0.246	0.085	0.008	0.068	0.004	0.083	0.009	0.004	0.202

S3.2 Adiabatic and Diabatic Minima

In order to investigate further the coupling of $A(L_a)$, $A(L_b)$ and their relative stability with respect $A(n_N\pi^*1)$ we computed the TD-DFT adiabatic states of the base pair at the minima of the $A(L_a)$, $A(L_b)$ and $A(n_N\pi^*1)$ diabatic states, as predicted by the $FrD(MM_{ref})$-LVC model. As shown in Figure S6, the minimum of $A(L_a)$ lies at 5.11 eV, very close to the predicted LVC adiabatic state from all three procedures (see Table 3 in the main manuscript). The minimum of $A(L_b)$ lies on the contrary at 5.40 eV, $\sim$ 0.1 eV above what predicted by the LVC models (which however shows that the adiabatic state is quite mixed). Finally $A(n_N\pi^*1)$ is predicted to lie at 5.03 eV, with a further moderate stabilization with respect to what predicted by St-LVC and $FrD(MM_{ref})$-LVC models, and a much more significant stabilization than with respect to FrD-LVC minimum. Starting from the geometries of these diabatic minima we also attempted a re-optimization of these three states with TD-DFT (data reported in Figure S7). We were able to locate both the $A(L_a)$ and $A(n_N\pi^*1)$ minima and found that they exhibit only a very slight stabilization ($\sim$ 0.03 eV) indicating that the minimum geometries estimated by LVC are quite accurate. The optimization algorithm failed to of optimize $A(L_b)$, ending instead in the $A(L_a)$ minimum.

	GS	Min of Diabatic state La	Min of Diabatic state Lb	Min of Diabatic state $n_N$pi*
State 3 (La)	5.45 eV<br>f=0.1258<br><br>76 -79 0.48<br>76 -78 0.42<br>73 -78 -0.23	5.11 eV<br>f=0.2886<br><br>76 -78 0.65067<br>76 -79 -0.22293<br>73 -79 -0.10491	5.2802 eV<br>f=0.3521<br><br>76 -78 0.65976<br>76 -77 -0.15703<br>73 -79 0.12452<br>75 - 77 0.11244	5.475 eV<br>f=0.3261<br><br>76 -78 0.66031<br>76 -79 -0.14230<br>76 -77 0.13036<br>73 -79 -0.10490
State 4 (Lb)	5.56 eV<br>f=0.2030<br><br>75 - 77 -0.1<br>76 – 77 -0.13<br>76 -79 -0.36<br>76 - 78 0.51<br>73 - 78 0.2<br>73 – 79 0.1	5.51 eV<br>f=0.1311<br><br>75 – 77 -0.2999<br>76 -79 0.5271<br>76 - 78 0.1958<br>73 - 78 0.2265	5.3958 eV<br>f=0.0375<br><br>76 -79 0.63110<br>73 -78 -0.26105<br>76 -78 0.10845	5.99 eV<br>f=0.0662<br><br>76 -79 0.52142<br>73 -78 0.37870<br>74 -77 0.15585<br>76 -78 0.15116<br>76 -77 0.13821
State 5 ($n_N$pi*)	5.66 eV<br>f=0.0003<br><br>74 - 81 0.-0.12<br>74 - 78 0.64<br>74 – 79 0.15<br>72 - 78 -0.10	5.4369 eV<br>f=0.0001<br><br>74 - 78 0.67316<br>74 - 79 -0.11714	5.5845 eV<br>f=0.0001<br><br>72 -77 0.57546<br>74 -77 0.21390<br>68 -77 -0.21421<br>72 -80 0.16251<br>69 -77 0.11841	5.0298 eV<br>f=0.0003<br><br>75 - 78 0.66997<br>75 - 77 0.13085<br>75 - 81 0.11458

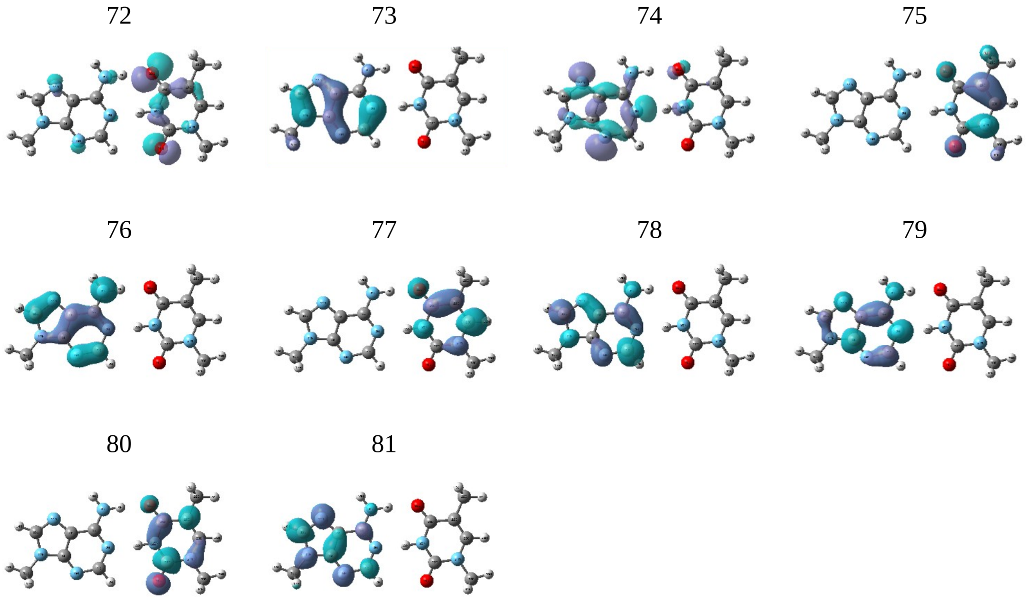

Figure S6: Adiabatic states energies and character predicted by TD-DFT in the minima of the lowest-energy diabatic states localized on A predicted by the FrD(MM_{ref})-LVC 12-state model. Notice that the MOs identified by a numeric label are practically identical when computed in the ground state minimum and in all minima. The only exception is MO 74 at the ground state minimum which is equal to MO 75 in A($n_N\pi^*1$) minimum.

	GS	Min of Adiabatic state La	Min of Adiabatic state Lb	Min of Adiabatic state $n_N$pi*
State 3 (La)	5.45 eV<br>f=0.1311<br><br>76 -79 0.48<br>76 -78 0.42<br>73 -78 -0.23	5.08 eV<br>f=0.3226<br><br>76 -78 -0.67231<br>76 -79 0.16088	We reach to the minimum of La	5.5852eV<br>f=0.2958<br><br>76 -78 -0.65523<br>76 -79 0.15168<br>76 -77 -0.13813<br>73 -79 0.10237<br>73 -78 0.10595
State 4 (Lb)	5.56 eV<br>f=0.1721<br><br>75 - 77 -0.1<br>76 – 77 -0.13<br>76 -79 -0.36<br>76 - 78 0.51<br>73 - 78 0.2<br>73 – 79 0.1	5.6241 eV<br>f=0.0982<br><br>75 – 77 0.26571<br>76 -79 -0.54817<br>76 - 78 -0.13151<br>73 - 78 -0.24274<br>76 -77 -0.19944		6.1849 eV<br>f=0.0662<br><br>76 -79 -0.50679<br>73 -78 -0.37893<br>74 -77 -0.17392<br>76 -78 -0.15785<br>76 -77 -0.15691
State 5 ($n_N$pi*)	5.66 eV<br>f=0.0001<br><br>74 - 81 0.-0.12<br>74 - 78 0.64<br>74 – 79 0.15<br>72 - 78 -0.10	5.4591 eV<br>f=0.0001<br><br>74 - 78 -0.67589		4.9941 eV<br>f=0.0002<br><br>75 - 78 -0.66855<br>75 - 77 -0.14265<br>75 - 81 -0.10880

Figure S7: Energies of the lowest-energy planar adiabatic excited state minima mostly localized on A from TD-DFT excited state geometry optimisations. It is noteworthy that the TD-DFT adiabatic states energies of L_a, L_b and $n_N\pi^*1$ obtained either in the diabatic minima or after a TD-DFT optimization are very similar (Compare this with Figure S6.)

Table S8: Eigenvalues (eV) of A' adiabatic LVC states in the predicted diabatic minima, and the corresponding normalised eigenvectors. From 12-state Hamiltonians parametrized by CAM-B3LYP functional and 6-31G(d) basis set.

In Min Diab<br>State →	T<br>$\pi\pi^*1$	A<br>L_a	A<br>L_b	A→T<br>CT	T<br>$\pi\pi^*2$	T<br>$\pi\pi^*3$	A<br>$\pi\pi^*3$
				St-LVC			
Eigenval. (eV) →<br>Eigenvec. ↓	4.99	5.16	5.33	5.00	6.13	6.33	6.69
1 T($\pi\pi^*1$)	0.991	0.132	0.350	-0.103	-0.121	-0.027	-0.017
2 T($n_O\pi^*1$)	0.000	0.000	0.000	0.000	0.000	0.000	0.000
3 A(L_a)	-0.057	0.968	0.449	0.043	0.100	-0.037	0.016
4 A(L_b)	-0.111	0.175	0.819	-0.180	-0.461	-0.034	-0.006
5 A($n_N\pi^*1$)	0.000	0.000	0.000	0.000	0.000	0.000	0.000
6 A→T(CT)	-0.013	0.016	0.024	0.973	-0.357	-0.008	-0.002
7 A($n_N\pi^*2$)	0.000	0.000	0.000	0.000	0.000	0.000	0.000
8 T($n_O\pi^*2$)	0.000	0.000	0.000	0.000	0.000	0.000	0.000
9 A($n_N\pi^*3$)	0.000	0.000	0.000	0.000	0.000	0.000	0.000
10 T($\pi\pi^*2$)	0.000	0.053	0.003	-0.078	0.752	-0.057	-0.017
11 T($\pi\pi^*3$)	0.021	0.042	-0.018	-0.010	-0.053	0.930	-0.730
12 A($\pi\pi^*3$)	-0.005	0.094	-0.037	-0.012	-0.253	-0.358	0.682
				FrD(MM_{ref})-LVC			
Eigenval. (eV) →<br>Eigenvec. ↓	4.99	5.17	5.33	4.99	5.96	6.19	6.61
1 T($\pi\pi^*1$)	0.999	0.002	0.008	-0.035	-0.120	0.009	-0.001
2 T($n_O\pi^*1$)	0.000	0.000	0.000	0.000	0.000	0.000	0.000
3 A(L_a)	0.004	0.970	0.575	-0.008	0.006	0.003	0.086
4 A(L_b)	0.006	0.207	0.817	-0.014	0.009	0.008	-0.041
5 A($n_N\pi^*1$)	0.000	0.000	0.000	0.000	0.000	0.000	0.000
6 A→T(CT)	-0.006	0.000	0.002	0.999	-0.048	-0.000	0.004
7 A($n_N\pi^*2$)	0.000	0.000	0.000	0.000	0.000	0.000	0.000
8 T($n_O\pi^*2$)	0.000	0.000	0.000	0.000	0.000	0.000	0.000
9 A($n_N\pi^*3$)	0.000	0.000	0.000	0.000	0.000	0.000	0.000
10 T($\pi\pi^*2$)	-0.002	0.000	-0.000	0.014	0.989	-0.046	-0.015
11 T($\pi\pi^*3$)	0.021	0.001	0.001	0.000	0.062	0.998	0.001
12 A($\pi\pi^*3$)	-0.000	0.120	-0.037	0.003	-0.000	-0.000	0.995
				FrD-LVC			
Eigenval. (eV) →<br>Eigenvec. ↓	5.00	5.19	5.36	5.01	5.97	6.21	6.62
1 T($\pi\pi^*1$)	0.999	0.002	0.007	-0.036	-0.063	0.048	-0.001
2 T($n_O\pi^*1$)	0.000	0.000	0.000	0.000	0.000	0.000	0.000
3 A(L_a)	0.004	0.965	0.661	-0.009	0.008	0.002	0.072
4 A(L_b)	0.004	0.231	0.749	-0.012	0.009	0.003	-0.025
5 A($n_N\pi^*1$)	0.000	0.000	0.000	0.000	0.000	0.000	0.000
6 A→T(CT)	-0.005	0.000	0.002	0.999	-0.062	0.000	0.001
7 A($n_N\pi^*2$)	0.000	0.000	0.000	0.000	0.000	0.000	0.000
8 T($n_O\pi^*2$)	0.000	0.000	0.000	0.000	0.000	0.000	0.000
9 A($n_N\pi^*3$)	0.000	0.000	0.000	0.000	0.000	0.000	0.000
10 T($\pi\pi^*2$)	-0.009	0.000	-0.000	0.013	0.987	-0.125	-0.015
11 T($\pi\pi^*3$)	0.020	0.001	0.001	0.001	0.128	0.990	0.000
12 A($\pi\pi^*3$)	-0.000	0.123	-0.028	0.002	-0.000	-0.000	0.996

Table S9: Eigenvalues (eV) of A" adiabatic LVC states in the predicted diabatic minima, and the corresponding normalised eigenvectors. From 12-state Hamiltonians parametrized by CAM-B3LYP functional and 6-31G(d) basis set.

In Min Diab	T	A	A	T	A
State →	$n_O\pi^*1$	$n_N\pi^*1$	$n_N\pi^*2$	$n_O\pi^*2$	$n_N\pi^*3$
			St-LVC		
Eigenval. (eV) →	4.97	5.08	5.66	5.90	6.24
Eigenvec. ↓					
1 T($\pi\pi^*1$)	0.000	0.000	0.000	0.000	0.000
2 T($n_O\pi^*1$)	0.999	-0.011	0.158	0.601	0.002
3 A(L_a)	0.000	0.000	0.000	0.000	0.000
4 A(L_b)	0.000	0.000	0.000	0.000	0.000
5 A($n_N\pi^*1$)	0.010	0.990	-0.625	0.000	0.207
6 A→T(CT)	0.000	0.000	0.000	0.000	0.000
7 A($n_N\pi^*2$)	-0.014	0.132	0.761	0.017	0.396
8 T($n_O\pi^*2$)	-0.027	-0.002	-0.011	0.798	-0.012
9 A($n_N\pi^*3$)	0.004	0.035	0.059	0.006	0.894
10 T($\pi\pi^*2$)	0.000	0.000	0.000	0.000	0.000
11 T($\pi\pi^*3$)	0.000	0.000	0.000	0.000	0.000
12 A($\pi\pi^*3$)	0.000	0.000	0.000	0.000	0.000
			FrD(MM_{ref})-LVC		
Eigenval. (eV) →	4.96	5.12	5.65	5.86	6.25
Eigenvec. ↓					
1 T($\pi\pi^*1$)	0.000	0.000	0.000	0.000	0.000
2 T($n_O\pi^*1$)	0.999	0.001	0.009	-0.672	-0.001
3 A(L_a)	0.000	0.000	0.000	0.000	0.000
4 A(L_b)	0.000	0.000	0.000	0.000	0.000
5 A($n_N\pi^*1$)	-0.001	0.989	-0.676	-0.001	0.202
6 A→T(CT)	0.000	0.000	0.000	0.000	0.000
7 A($n_N\pi^*2$)	-0.000	0.144	0.734	-0.002	0.324
8 T($n_O\pi^*2$)	-0.024	-0.000	0.000	0.739	-0.000
9 A($n_N\pi^*3$)	-0.001	0.030	0.056	-0.000	0.924
10 T($\pi\pi^*2$)	0.000	0.000	0.000	0.000	0.000
11 T($\pi\pi^*3$)	0.000	0.000	0.000	0.000	0.000
12 A($\pi\pi^*3$)	0.000	0.000	0.000	0.000	0.000
			FrD-LVC		
Eigenval. (eV) →	4.97	5.21	5.74	5.89	6.23
Eigenvec. ↓					
1 T($\pi\pi^*1$)	0.000	0.000	0.000	0.000	0.000
2 T($n_O\pi^*1$)	0.999	0.003	0.008	-0.611	-0.001
3 A(L_a)	0.000	0.000	0.000	0.000	0.000
4 A(L_b)	0.000	0.000	0.000	0.000	0.000
5 A($n_N\pi^*1$)	-0.001	0.991	-0.611	0.001	0.193
6 A→T(CT)	0.000	0.000	0.000	0.000	0.000
7 A($n_N\pi^*2$)	-0.000	0.115	0.789	-0.000	0.003
8 T($n_O\pi^*2$)	0.023	0.000	-0.000	0.790	-0.000
9 A($n_N\pi^*3$)	-0.001	0.063	0.050	0.000	0.981
10 T($\pi\pi^*2$)	0.000	0.000	0.000	0.000	0.000
11 T($\pi\pi^*3$)	0.000	0.000	0.000	0.000	0.000
12 A($\pi\pi^*3$)	0.000	0.000	0.000	0.000	0.000

Table S10: Energies E_{ii}^{D} of the diabatic states i on all predicted diabatic minima of AT in C_{s} symmetry in gas phase obtained from different LVC models with 12 states and CAM-B3LYP/6-31G(d) parametrization.

IN MIN STATE ↓	T ($\pi\pi^*1$)	T ($n_O\pi^*1$)	A (L_a)	A (L_b)	A ($n_N\pi^*1$)	A→T (CT)	A ($n_N\pi^*2$)	T ($n_O\pi^*2$)	A ($n_N\pi^*3$)	T ($\pi\pi^*2$)	T ($\pi\pi^*3$)	A ($\pi\pi^*3$)
						St-LVC						
S_0	5.322	5.359	5.454	5.562	5.663	6.075	6.140	6.439	6.610	6.625	6.725	6.807
T($\pi\pi^*1$)	**4.993**	5.369	5.749	5.837	6.004	5.756	6.489	6.585	6.941	6.483	6.903	7.021
T($n_O\pi^*1$)	5.396	**4.966**	5.848	5.944	6.025	5.955	6.516	6.716	7.003	6.385	7.251	7.111
A(L_a)	5.586	5.658	**5.156**	5.544	5.520	5.987	6.227	6.743	6.834	6.905	7.010	7.032
A(L_b)	5.499	5.579	5.369	**5.332**	5.721	5.978	6.196	6.654	6.627	6.798	6.928	6.914
A($n_N\pi^*1$)	5.916	5.909	5.595	5.971	**5.081**	6.433	6.214	7.060	6.757	7.193	7.306	7.296
A→T(CT)	5.746	5.917	6.140	6.307	6.512	**5.003**	7.183	7.229	7.600	6.890	8.057	7.808
A($n_N\pi^*2$)	5.821	5.821	5.722	5.867	5.635	6.525	**5.661**	6.943	6.857	7.067	7.190	7.208
T ($n_O\pi^*2$)	5.720	5.823	6.042	6.128	6.283	6.373	6.745	**5.858**	7.216	6.953	7.044	7.282
A($n_N\pi^*3$)	5.693	5.727	5.749	5.717	5.597	6.362	6.277	6.833	**6.241**	6.977	7.069	6.997
T ($\pi\pi^*2$)	5.339	5.214	5.924	5.993	6.137	5.756	6.590	6.674	7.080	**6.137**	7.560	7.171
T($\pi\pi^*3$)	5.560	5.881	5.830	5.924	6.051	6.724	6.514	6.566	6.973	7.361	**6.336**	7.072
A($\pi\pi^*3$)	5.314	5.377	5.489	5.547	5.678	6.111	6.170	6.441	6.539	6.609	6.709	**6.699**
						FrD(MM_{ref})-LVC						
S_0	5.336	5.406	5.460	5.571	5.675	6.135	6.150	6.444	6.624	6.658	6.740	6.771
T($\pi\pi^*1$)	**4.988**	5.414	5.795	5.915	6.044	5.828	6.524	6.607	6.980	6.428	6.896	7.118
T($n_O\pi^*1$)	5.441	**4.961**	5.907	6.021	6.088	6.034	6.583	6.771	7.068	6.312	7.344	7.221
A(L_a)	5.615	5.700	**5.168**	5.536	5.527	5.980	6.239	6.746	6.800	6.963	7.022	6.958
A(L_b)	5.569	5.648	5.371	**5.334**	5.750	6.059	6.208	6.678	6.638	6.898	6.973	6.824
A($n_N\pi^*1$)	5.911	5.928	5.574	5.963	**5.121**	6.442	6.169	7.041	6.748	7.217	7.311	7.210
A→T(CT)	5.825	6.004	6.157	6.402	6.572	**4.991**	7.298	7.281	7.630	6.811	8.193	7.954
A($n_N\pi^*2$)	5.864	5.896	5.759	5.894	5.643	6.641	**5.648**	6.970	6.901	7.136	7.260	7.140
T ($n_O\pi^*2$)	5.735	5.872	6.054	6.152	6.302	6.412	6.758	**5.860**	7.231	6.919	7.001	7.362
A($n_N\pi^*3$)	5.715	5.776	5.716	5.719	5.616	6.368	6.296	6.838	**6.253**	7.034	7.138	6.915
T ($\pi\pi^*2$)	5.457	5.315	6.173	6.273	6.379	5.844	6.825	6.821	7.329	**5.958**	7.951	7.478
T($\pi\pi^*3$)	5.686	6.107	5.993	6.109	6.235	6.987	6.711	6.664	7.194	7.712	**6.197**	7.310
A($\pi\pi^*3$)	5.495	5.572	5.516	5.548	5.721	6.334	6.178	6.611	6.558	6.826	6.897	**6.610**
						FrD-LVC						
S_0	5.347	5.415	5.475	5.577	5.755	6.157	6.199	6.450	6.587	6.655	6.737	6.762
T($\pi\pi^*1$)	**4.999**	5.420	5.810	5.922	6.117	5.866	6.570	6.615	6.943	6.433	6.873	7.106
T($n_O\pi^*1$)	5.454	**4.966**	5.925	6.031	6.179	6.065	6.650	6.775	7.030	6.345	7.315	7.217
A(L_a)	5.618	5.699	**5.191**	5.539	5.661	5.985	6.300	6.744	6.673	6.952	7.013	6.915
A(L_b)	5.562	5.637	5.370	**5.360**	5.853	6.090	6.281	6.666	6.555	6.874	6.950	6.778
A($n_N\pi^*1$)	5.911	5.939	5.647	6.007	**5.206**	6.438	6.230	7.043	6.706	7.210	7.327	7.205
A→T(CT)	5.851	6.017	6.162	6.436	6.630	**5.014**	7.323	7.290	7.570	6.811	8.165	7.918
A($n_N\pi^*2$)	5.832	5.879	5.754	5.903	5.698	6.599	**5.737**	6.935	6.831	7.096	7.230	7.076
T ($n_O\pi^*2$)	5.728	5.854	6.048	6.138	6.362	6.417	6.785	**5.887**	7.166	6.879	6.990	7.330
A($n_N\pi^*3$)	5.714	5.767	5.636	5.687	5.684	6.356	6.340	6.825	**6.228**	7.018	7.115	6.933
T ($\pi\pi^*2$)	5.459	5.336	6.169	6.260	6.442	5.850	6.860	6.792	7.272	**5.974**	7.893	7.448
T($\pi\pi^*3$)	5.655	6.064	5.987	6.093	6.316	6.962	6.750	6.660	7.127	7.650	**6.217**	7.278
A($\pi\pi^*3$)	5.490	5.567	5.491	5.523	5.796	6.317	6.198	6.602	6.546	6.806	6.880	**6.615**

S3.3 Dynamics

S3.3.1 Reference results for the dynamics of 1methyl-Thymine and 9methyl-Adenine

Figure S8 reports the dynamics of the electronic populations of the isolated nucleobases obtained with St-LVC Hamiltonians parametrized with CAM-B3LYP/-6-31G(d) calculations, the same level of theory adopted for AT in the main text. These results should be compared with those for AT reported in Figure 4 of the main text.

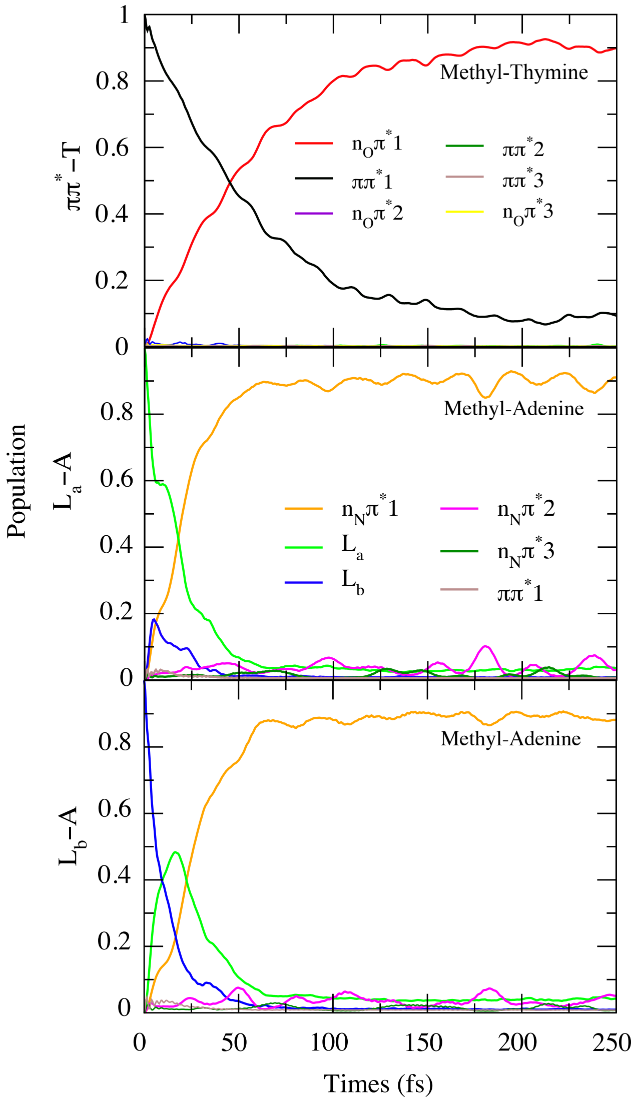

Figure S8: Diabatic state populations for 1-methylthymine following initial excitation to T($\pi\pi^*1$) (top), and for 9-methyladenine following initial excitation to A(L_a) (middle) and A(L_b) (bottom). For both bases, 6 state St-LVC models are utilised, parametrized by CAM-B3LYP/6-31G(d).

S3.3.2 Population of CT: AT vs GC

In both AT and GC the reorganization energy of CT state is about 1.15 eV. The CT state is energetically closer to other bright excited states in GC than AT, with CAM-B3LYP/6-31G(d) predicting the CT state to be ≈0.2 eV more stable than the lowest bright states for GC, whilst it is ≈0.7 eV less stable than the lowest bright states for AT, as shown in Table S12. Moreover, Table S11 shows that diabatic electronic coupling of the G→C(CT) state with G(L_a) is much greater than the coupling of the A→T(CT) state with A(L_a). Furthermore, the sum of the couplings for the $\pi\pi^*$ states of G and C to the G→C(CT) state is greater than the sum of the couplings for the $\pi\pi^*$ states of A and T to the A→T(CT) state.

Table S11: $\pi\pi^*$-CT couplings between diabatic states at the FC point ($E^{\mathrm{D}}_{ij}(0)$, eV) for AT and GC pairs, predicted via FrD-LVC and FrD(MM_{ref})-LVC models parametrized by CAM-B3LYP/6-31G(d). FrD-LVC data for GC previously published in ref. S5.

GC		AT	
FrD-LVC			
G(L_a) : G→C(CT)	0.065	A(L_a) : A→T(CT)	-0.007
G(L_b) : G→C(CT)	0.038	A(L_b) : A→T(CT)	0.061
C($\pi\pi^*1$) : G→C(CT)	0.022	T ($\pi\pi^*1$) : A→T(CT)	0.016
FrD(MM_{ref})-LVC			
G(L_a) : G→C(CT)	0.074	A(L_a) : A→T(CT)	-0.015
G(L_b) : G→C(CT)	-0.018	A(L_b) : A→T(CT)	0.062
C($\pi\pi^*1$) : G→C(CT)	0.031	T ($\pi\pi^*1$) : A→T(CT)	0.014

Table S12: Energies (eV) of the diabatic states of AT and GC computed with the FrD-LVC 12-state model, $E^{\mathrm{D}}_{ii}(0)$, as well as diabatic energies at predicted diabatic excited state planar minima (D_{min}) and adiabatic energies at the FC point from TD-DFT. All computations parametrized by CAM-B3LYP/6-31G(d).

GC				AT			
Diab. State	$E^{\mathrm{D}}_{ii}(0)$	$E^{\mathrm{D}}_{ii}[D_{min}]$	TD-DFT	Diab. State	$E^{\mathrm{D}}_{ii}(0)$	$E^{\mathrm{D}}_{ii}[D_{min}]$	TD-DFT
G→C(CT)1	5.17	3.94	5.07 (S_1)	A→T(CT)1	6.16	5.01	6.08(S_6)
C($\pi\pi^*1$)	5.33	4.94	5.27 (S_2)	T ($\pi\pi^*1$)	5.35	5.00	5.32(S_1)
G(L_a)	5.42	5.09	5.31 (S_3)	A(L_a)	5.47	5.19	5.45(S_3)

S3.4 Time-dependence of diabatic potentials

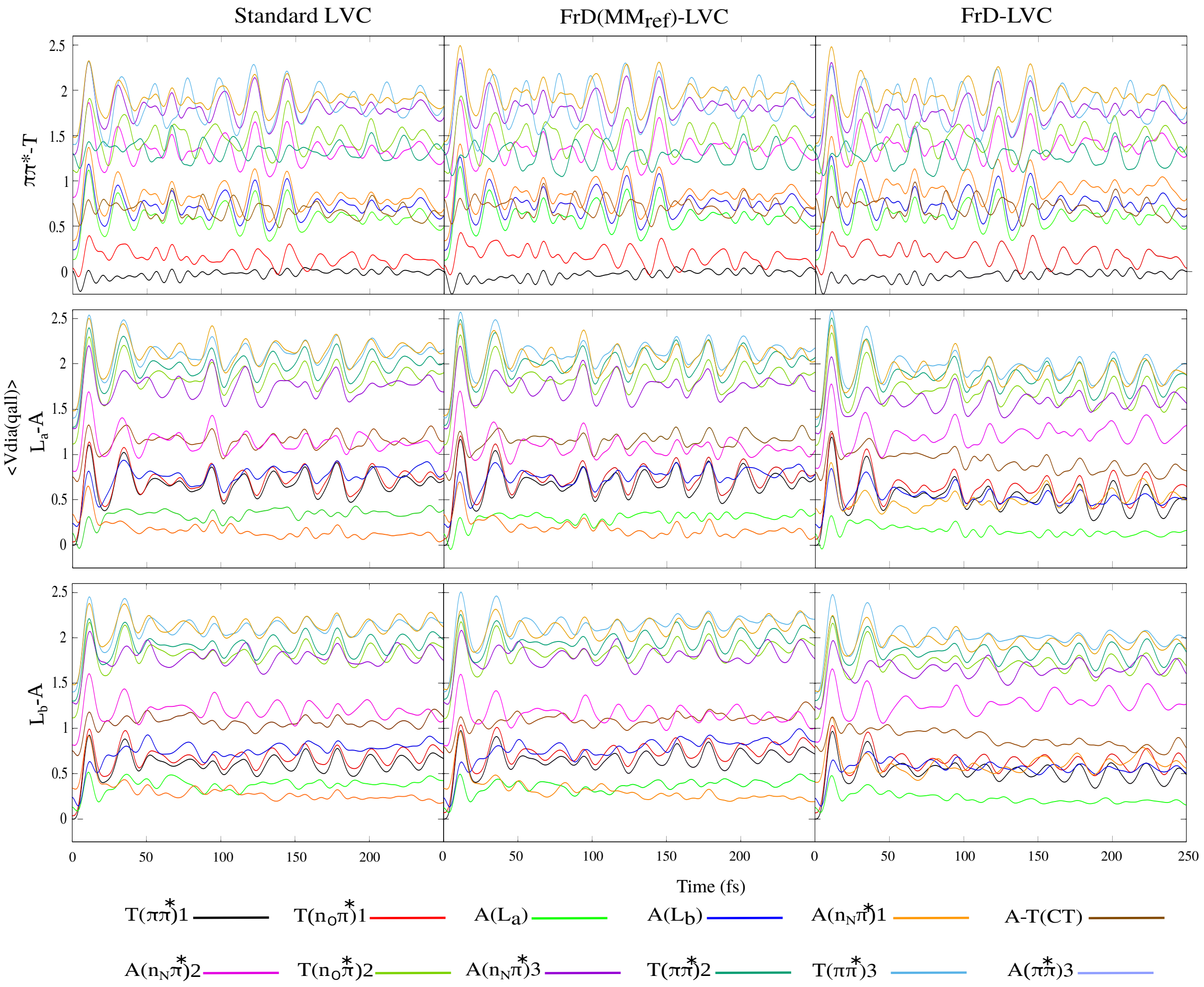

Figure S9: Expectation of diabatic PES for AT with initial excitation of T($\pi\pi^{*}1$) (top), A(L_a) (middle) and A(L_b) (bottom) obtained by St-LVC(left), FrD(MM_{ref})-LVC(middle) FrD-LVC(right).This figure is an extended version of Figure 5 in the main text since it reports the diabatic potentials for all 12 states.

S3.5 Study of the convergence of the electronic population dynamics with the number of diabatic states

In the Section "The excited states at the FC position" in the main text we explain how we built up 12-state models for FrD(MM_{ref})-LVC and FrD-LVC calculations. In practice, we first selected a few states considered "chemically relevant" on the grounds of what is known about the photophysics of the two nucleobases and the dimer (the lowest bright $\pi\pi^*$ and nπ^* states of A and T and the lowest A$\rightarrow$T CT state) and we checked that such fragment states were actually involved in the formation of the lowest TD-DFT states for the AT dimer at the FC position. Afterward, we completed the set of 12 states including further fragment states defined to be as similar as possible to other adiabatic states of the dimer in the FC position, that are either low-lying or coupled to those already selected (according to the st-LVC model).

In order to analyse the convergence of our calculations with respect to the dimension of the diabatic basis set we built up 16-states and 22-states models. In doing that, we could follow exactly the same strategy adopted for the 12-states model, with an exception for 22-state case: in fact TD-DFT state 19 (i.e. the 19th state in the St-LVC model) is actually made up by two opposite CT states, a A$\rightarrow$T component and a T$\rightarrow$A component, so that a one-to-one correspondence with a single fragment diabatic state is impossible. Therefore, in order to make sure to have a diabatic basis set able to describe all the states included in the 22-state St-LVC model, for FrD(MM_{ref})-LVC and FrD-LVC in place of state 19 of the St-LVC model, we introduced two different fragment states, i.e. those corresponding to the two CT components, obtaining, "rigorously speaking", 23-state models. However, as it is shown in the following Tables, the diabatic state T$\rightarrow$A (CT2) is predicted to be very high in energy and, consequently, it has a practically negligible effect on the dynamics. For these reasons, for the sake of brevity in the main text we use the label "22-state" also for these FrD(MM_{ref})-LVC and FrD-LVC models.

Increasing further number of states, the St-LVC states are too mixed and delocalized and therefore it is not possible anymore to establish a one-to-one correspondence with the FrD(MM_{ref})-LVC and FrD-LVC states. Therefore in order to push further the analysis of the convergence of

the results with the dimension of the diabatic basis set, we focused on the St-LVC model only, and we performed a benchmark calculation adopting as reference states for the diabatic states all the 32 lowest-energy TD-DFT states of AT at the FC position.

S3.5.1 FrD(MM_{ref})-LVC calculations

Table S13: Energies of adiabatic LVC states and their corresponding diabatic character from FrD(MM_{ref})-LVC models with different numbers of diabatic states, as well as TD-DFT energies for states with similar characters. Obtained at the CAM-B3LYP/6-31G(d) level.

Character	10 St	12 St	16 St	17 St	22 St	TD-DFT
1.T($\pi\pi^*$1)	5.33	5.33	5.32	5.32	5.32	5.32
2.T($n_O\pi^*$1)	5.41	5.41	5.41	5.41	5.39	5.36
3.A(L_a)	5.46	5.46	5.46	5.46	5.46	5.45
4.A(L_b)	5.57	5.57	5.57	5.57	5.57	5.56
5.A($n_N\pi^*$1)	5.67	5.67	5.67	5.67	5.69	5.66
6.A→T(CT1)	6.14	6.14	6.14	6.14	6.13	6.08
7.A($n_N\pi^*$2)	6.15	6.15	6.15	6.15	6.15	6.14
8.T($n_O\pi^*$2)	6.44	6.44	6.44	6.44	6.44	6.44
9.A($n_N\pi^*$3)	6.63	6.63	6.63	6.63	6.63	6.61
10.T($\pi\pi^*$2)	6.67	6.64	6.64	6.64	6.64	6.62
11.T($\pi\pi^*$3)		6.73	6.73	6.73	6.73	6.73
12.A($\pi\pi^*$3)		6.81	6.81	6.81	6.81	6.81
13.T($n_O\pi^*$3)			7.05	7.05	6.99	6.89
14.A($\pi\pi^*$4)			7.07	7.07	7.07	7.06
15.A($\pi\pi^*$5)			7.13	7.13	7.13	7.13
16.A($n_N\pi^*$4)			7.17	7.17	7.18	7.16
17.T→A(CT1)				7.27	7.27	7.27
18.A($n_N\pi^*$5)					7.36	7.32
19.T→A(CT2)					9.15	
20.A→T(CT2)					7.49	7.35
21.A→T(CT3)					7.42	7.39
22.A($\pi\pi^*$6)					7.49	7.46
23.T($n_O\pi^*$4)					7.68	7.56

Table S14: Norm of coupling vector $\sqrt{\boldsymbol{\lambda}_{ij} \cdot \boldsymbol{\lambda}_{ij}}$ for 22 state FrD(MM_{ref})-LVC model of AT in C_s symmetry at ground state equilibrium geometry, parametrized with CAM-B3LYP/6-31G(d).

	1	2	3	4	5	6	7	8	9	10	11	12
1.T($\pi\pi^*$1)	0.344											
2.T($n_O\pi^*$1)	0.060	0.418										
3.A(L_a)	0.004	0.003	0.317									
4.A(L_b)	0.005	0.008	0.150	0.278								
5.A($n_N\pi^*$1)	0.002	0.000	0.105	0.067	0.417							
6.A→T(CT1)	0.006	0.044	0.007	0.012	0.001	0.605						
7.A($n_N\pi^*$2)	0.002	0.001	0.055	0.080	0.228	0.001	0.369					
8.T($n_O\pi^*$2)	0.040	0.098	0.002	0.002	0.000	0.005	0.000	0.494				
9.A($n_N\pi^*$3)	0.002	0.001	0.082	0.114	0.222	0.001	0.105	0.000	0.319			
10.T($\pi\pi^*$2)	0.111	0.076	0.004	0.008	0.005	0.011	0.009	0.026	0.005	0.503		
11.T($\pi\pi^*$3)	0.186	0.036	0.003	0.004	0.002	0.001	0.002	0.080	0.002	0.063	0.420	
12.A($\pi\pi^*$3)	0.004	0.006	0.184	0.242	0.077	0.007	0.071	0.004	0.085	0.009	0.004	0.212
13.T($n_O\pi^*$3)	0.106	0.125	0.001	0.003	0.001	0.015	0.001	0.215	0.001	0.086	0.078	0.005
14.A($\pi\pi^*$4)	0.006	0.008	0.128	0.141	0.079	0.003	0.047	0.004	0.074	0.008	0.005	0.136
15.A($\pi\pi^*$5)	0.004	0.005	0.198	0.173	0.040	0.005	0.032	0.002	0.052	0.005	0.003	0.124
16.A($n_N\pi^*$4)	0.002	0.001	0.116	0.072	0.150	0.002	0.133	0.001	0.192	0.007	0.003	0.078
17.T→A(CT1)	0.009	0.001	0.005	0.005	0.011	0.000	0.003	0.001	0.012	0.002	0.008	0.004
18.A($n_N\pi^*$5)	0.001	0.002	0.073	0.119	0.120	0.002	0.189	0.001	0.192	0.009	0.002	0.087
19.T→A(CT2)	0.005	0.006	0.023	0.020	0.012	0.001	0.044	0.011	0.006	0.007	0.003	0.021
20.A→T(CT2)	0.011	0.027	0.001	0.003	0.007	0.129	0.012	0.013	0.002	0.035	0.004	0.007
21.A→T(CT3)	0.002	0.015	0.006	0.008	0.000	0.219	0.001	0.015	0.000	0.002	0.008	0.005
22.A($\pi\pi^*$6)	0.004	0.003	0.162	0.151	0.055	0.001	0.089	0.003	0.070	0.009	0.003	0.236
23.T($n_O\pi^*$4)	0.051	0.235	0.002	0.006	0.000	0.012	0.001	0.133	0.000	0.048	0.088	0.005

	13	14	15	16	17	18	19	20	21	22	23
13.T($n_O\pi^*$3)	0.540										
14.A($\pi\pi^*$4)	0.005	0.238									
15.A($\pi\pi^*$5)	0.002	0.142	0.296								
16.A($n_N\pi^*$4)	0.000	0.111	0.078	0.220							
17.T→A(CT1)	0.001	0.006	0.003	0.009	0.458						
18.A($n_N\pi^*$5)	0.001	0.037	0.060	0.216	0.006	0.309					
19.T→A(CT2)	0.011	0.015	0.005	0.040	0.004	0.043	0.556				
20.A→T(CT2)	0.035	0.002	0.003	0.003	0.000	0.004	0.001	0.608			
21.A→T(CT3)	0.004	0.003	0.004	0.002	0.000	0.001	0.001	0.006	0.537		
22.A($\pi\pi^*$6)	0.003	0.221	0.090	0.057	0.003	0.109	0.018	0.010	0.001	0.228	
23.T($n_O\pi^*$4)	0.100	0.008	0.004	0.001	0.000	0.001	0.008	0.024	0.049	0.002	0.436

Table S15: Eigenvalues (eV) of A' adiabatic LVC states and corresponding normalised eigenvectors showing the contribution of the $\pi\pi^*$ and CT diabatic states from the 22-state FrD(MM$_{ref}$)-LVC model of AT in C_s symmetry at the FC point. Parametrized by CAM-B3LYP with 6-31G(d) basis set.

Adiabatic states→	S_1	S_3	S_4	S_6	S_{10}	S_{11}	S_{12}	S_{14}	S_{15}	S_{17}	S_{21}	S_{22}
Eigenval.(eV)→ Eigenvec. ↓	5.3241	5.4561	5.5657	6.1255	6.6396	6.7261	6.8090	7.0718	7.1311	7.2716	7.4180	7.4846
1.T($\pi\pi^*$1)	0.980	-0.077	-0.170	0.010	-0.011	-0.015	0.028	0.029	0.013	0.014	-0.005	0.000
3.A(L_a)	0.093	0.989	0.087	-0.024	-0.012	-0.035	-0.031	-0.013	0.014	0.007	0.005	0.003
4.A(L_b)	0.159	-0.097	0.973	0.120	0.048	-0.008	0.006	0.024	0.003	0.003	-0.001	-0.005
6.A→T(CT1)	-0.027	0.034	-0.107	0.982	-0.112	-0.012	-0.017	-0.011	-0.008	0.002	-0.090	-0.003
10.T($\pi\pi^*$2)	0.011	0.002	-0.055	0.092	0.924	-0.101	-0.329	-0.093	-0.042	-0.004	0.008	0.058
11.T($\pi\pi^*$3)	0.027	0.015	0.009	0.004	-0.040	0.916	-0.392	0.005	0.030	0.009	0.033	0.042
12.A($\pi\pi^*$3)	-0.017	0.050	-0.020	0.056	0.345	0.379	0.852	0.050	0.024	-0.003	0.004	-0.019
14.A($\pi\pi^*$4)	-0.028	0.016	-0.022	0.013	0.067	-0.031	-0.076	0.988	-0.015	-0.088	0.001	-0.030
15.A($\pi\pi^*$5)	-0.014	-0.013	-0.005	0.009	0.032	-0.040	-0.024	0.005	0.996	-0.046	-0.017	-0.013
17.T→A(CT1)	-0.019	-0.005	-0.003	-0.000	0.011	-0.012	-0.000	0.089	0.044	0.994	0.001	0.034
21.A→T(CT3)	0.001	-0.003	-0.009	0.089	-0.018	-0.033	0.011	-0.001	0.016	-0.002	0.995	0.005
22.A($\pi\pi^*$6)	-0.002	-0.002	0.006	-0.000	-0.043	-0.026	0.049	0.033	0.012	-0.037	-0.008	0.996

Table S16: Eigenvalues (eV) of A" adiabatic LVC states and corresponding normalised eigenvectors showing the contribution of the nπ^* diabatic states from the 22-state FrD(MM$_{\text{ref}}$)-LVC model of AT in C_s symmetry at the FC point. Parameterized by CAM-B3LYP with 6-31G(d) basis set.

Adiabatic states→	S_2	S_5	S_7	S_8	S_9	S_{13}	S_{16}	S_{18}	S_{20}	S_{23}	S_{19}
Eigenval.(eV)→ Eigenvec. ↓	5.3943	5.6858	6.1504	6.4424	6.6341	6.9904	7.1828	7.3634	7.4876	7.6869	9.1519
2.T($n_O\pi^*1$)	0.997	0.001	-0.012	-0.007	0.000	-0.013	0.000	0.002	-0.058	-0.028	-0.006
5.A($n_N\pi^*1$)	-0.001	0.996	-0.069	0.001	0.047	0.004	-0.015	-0.010	0.008	0.004	-0.008
7.A($n_N\pi^*2$)	0.008	0.071	0.992	-0.021	-0.041	-0.028	-0.022	0.017	-0.037	-0.016	-0.068
8.T($n_O\pi^*2$)	0.005	0.000	0.018	0.998	0.002	-0.019	0.005	-0.001	-0.035	-0.002	-0.014
9.A($n_N\pi^*3$)	0.000	-0.045	0.045	-0.002	0.996	0.002	-0.041	-0.040	-0.001	0.000	0.003
13.T($n_O\pi^*3$)	-0.008	-0.000	0.013	0.007	-0.002	0.946	0.009	0.012	-0.293	-0.126	-0.022
16.A($n_N\pi^*4$)	-0.000	0.017	0.024	-0.005	0.045	-0.010	0.990	0.112	0.000	-0.003	0.049
18.A($n_N\pi^*5$)	0.000	0.005	-0.020	0.002	0.036	0.002	-0.110	0.990	0.045	0.012	-0.055
19.T→A(CT2)	0.007	0.013	0.065	0.013	-0.006	0.017	-0.056	0.051	-0.008	-0.012	0.994
20.A→T(CT2)	0.067	-0.007	0.050	0.035	-0.003	0.317	0.008	-0.042	0.838	0.430	0.005
23.T($n_O\pi^*4$)	-0.001	0.000	-0.003	-0.013	-0.000	-0.019	0.002	0.009	-0.449	0.892	0.007

Table S17: Diabatic energies ($E^{\mathrm{D}}_{ii}(0)$) and electronic couplings ($E^{\mathrm{D}}_{ij}(0)$) of A' states from the FrD(MM_{ref})-LVC 22-state model of AT in C_{s} symmetry at ground state equilibrium geometry (eV).

STATE	T ($\pi\pi^*1$)	A (L_a)	A (L_b)	A→T (CT1)	T ($\pi\pi^*2$)	T ($\pi\pi^*3$)	A ($\pi\pi^*3$)	A ($\pi\pi^*4$)	A ($\pi\pi^*5$)	T→A (CT1)	A→T (CT2)	A ($\pi\pi^*6$)
1.T($\pi\pi^*1$)	5.336											
3.A(L_a)	-0.015	5.460										
4.A(L_b)	-0.037	0.004	5.570									
6.A→T(CT1)	0.014	-0.014	0.061	6.135								
10.T($\pi\pi^*2$)	-0.029	0.004	0.048	-0.052	6.659							
11.T($\pi\pi^*3$)	-0.036	-0.022	-0.015	-0.003	0.015	6.740						
12.A($\pi\pi^*3$)	0.027	-0.060	0.029	-0.037	-0.058	-0.027	6.771					
14.A($\pi\pi^*4$)	0.046	-0.019	0.041	-0.016	-0.038	0.004	0.008	7.067				
15.A($\pi\pi^*5$)	0.023	0.025	0.009	-0.009	-0.021	0.013	0.007	-0.003	7.129			
17.T→A(CT1)	0.035	0.014	0.011	0.000	-0.005	0.008	-0.001	-0.020	-0.008	7.270		
21.A→T(CT2)	-0.009	0.011	0.002	-0.116	0.002	0.023	0.001	-0.000	-0.006	0.000	7.406	
22.A($\pi\pi^*6$)	0.006	0.005	-0.011	-0.002	0.046	0.030	-0.008	-0.010	-0.004	0.007	-0.001	7.480

Table S18: Diabatic energies ($E_{ii}^{\mathrm{D}}(0)$) and electronic couplings ($E_{ij}^{\mathrm{D}}(0)$) of A" states from the FrD(MM_{ref})-LVC 22-state model of AT in C_s symmetry at ground state equilibrium geometry (eV).

STATE	T ($n_O\pi^*1$)	A ($n_N\pi^*1$)	A ($n_N\pi^*2$)	T ($n_O\pi^*2$)	A ($n_N\pi^*3$)	T ($n_O\pi^*3$)	A ($n_N\pi^*4$)	A ($n_N\pi^*5$)	T→A (CT2)	A→T (CT2)	T ($n_O\pi^*4$)
2.T($n_O\pi^*1$)	5.404										
5.A($n_N\pi^*1$)	-0.000	5.691									
7.A($n_N\pi^*2$)	-0.000	-0.033	6.167								
8.T($n_O\pi^*2$)	-0.003	0.000	-0.001	6.444							
9.A($n_N\pi^*3$)	-0.000	0.045	-0.019	-0.000	6.633						
13.T($n_O\pi^*3$)	0.025	-0.000	-0.000	0.002	0.000	7.045					
16.A($n_N\pi^*4$)	0.000	-0.026	-0.032	0.001	-0.025	0.000	7.188				
18.A($n_N\pi^*5$)	-0.000	-0.011	0.030	-0.000	-0.027	-0.000	0.014	7.366			
19.T→A(CT2)	-0.025	-0.033	-0.201	-0.040	0.009	-0.046	0.096	-0.098	9.126		
20.A→T(CT2)	-0.140	0.017	-0.063	-0.036	-0.001	-0.160	-0.001	0.007	-0.000	7.460	
23.T($n_O\pi^*4$)	-0.003	-0.000	-0.000	0.013	0.000	-0.014	-0.001	0.000	0.010	0.081	7.646

S3.5.2 FrD-LVC calculations

Table S19: Energies of adiabatic LVC states and their corresponding diabatic character from FrD-LVC models with different numbers of diabatic states, as well as TD-DFT energies for states with similar characters. Obtained at the CAM-B3LYP/6-31G(d) level.

State	Character	10 St	12 St	16 St	17 St	19 St	22 St	TD-DFT
1	T($\pi\pi^*$1)	5.33	5.33	5.32	5.32	5.32	5.33	5.32
2	T($n_O\pi^*$1)	5.41	5.41	5.41	5.41	5.41	5.40	5.36
3	A(L_a)	5.48	5.46	5.46	5.46	5.46	5.46	5.45
4	A(L_b)	5.57	5.57	5.57	5.57	5.57	5.57	5.56
5	A($n_N\pi^*$1)	5.67	5.68	5.67	5.67	5.67	5.69	5.66
6	A→T(CT1)	6.16	6.16	6.16	6.16	6.15	6.14	6.08
7	A($n_N\pi^*$2)	6.21	6.21	6.17	6.17	6.16	6.16	6.14
8	T($n_O\pi^*$2)	6.45	6.45	6.45	6.45	6.45	6.44	6.44
9	A($n_N\pi^*$3)	6.66	6.65	6.64	6.64	6.63	6.64	6.61
10	T($\pi\pi^*$2)	6.67	6.66	6.65	6.65	6.64	6.64	6.62
11	T($\pi\pi^*$3)		6.73	6.73	6.73	6.73	6.73	6.73
12	A($\pi\pi^*$3)		6.82	6.81	6.81	6.81	6.81	6.81
13	T($n_O\pi^*$3)			7.08	7.08	7.08	6.95	6.89
14	A($\pi\pi^*$4)			7.09	7.09	7.09	7.07	7.06
15	A($\pi\pi^*$5)			7.13	7.13	7.13	7.13	7.13
16	A($n_N\pi^*$4)			7.18	7.18	7.17	7.19	7.16
17	T→A(CT1)					7.28	7.28	7.27
18	A($n_N\pi^*$5)					7.37	7.37	7.32
19	T→A(CT2)						9.12	7.35
20	A→T(CT2)						7.41	
21	A→T(CT3)						7.42	7.39
22	A($\pi\pi^*$6)				7.49	7.49	7.49	7.46
23	T($\pi\pi^*$4)						7.69	7.56

Table S20: Norm of coupling vector $\sqrt{\boldsymbol{\lambda}_{ij} \cdot \boldsymbol{\lambda}_{ij}}$ for 22 state FrD-LVC model of AT in C_{s} symmetry at ground state equilibrium geometry, parametrized with CAM-B3LYP/6-31G(d).

	1	2	3	4	5	6	7	8	9	10	11	12
1.T($\pi\pi^*$1)	0.347											
2.T($n_O\pi^*$1)	0.058	0.424										
3.A(L_a)	0.004	0.004	0.314									
4.A(L_b)	0.005	0.007	0.142	0.263								
5.A($n_N\pi^*$1)	0.002	0.001	0.097	0.062	0.422							
6.A→T(CT1)	0.006	0.043	0.006	0.011	0.001	0.607						
7.A($n_N\pi^*$2)	0.002	0.001	0.042	0.066	0.241	0.002	0.361					
8.T($n_O\pi^*$2)	0.041	0.105	0.002	0.002	0.000	0.005	0.001	0.483				
9.A($n_N\pi^*$3)	0.002	0.001	0.103	0.128	0.212	0.001	0.070	0.000	0.312			
10.T($\pi\pi^*$2)	0.104	0.075	0.004	0.008	0.006	0.011	0.011	0.024	0.004	0.496		
11.T($\pi\pi^*$3)	0.189	0.039	0.003	0.004	0.003	0.002	0.003	0.081	0.002	0.108	0.412	
12.A($\pi\pi^*$3)	0.004	0.006	0.207	0.247	0.087	0.008	0.067	0.004	0.084	0.009	0.004	0.202
13.T($n_O\pi^*$3)	0.104	0.113	0.001	0.002	0.001	0.017	0.001	0.198	0.000	0.094	0.073	0.004
14.A($\pi\pi^*$4)	0.007	0.009	0.123	0.133	0.086	0.003	0.055	0.004	0.083	0.008	0.006	0.140
15.A($\pi\pi^*$5)	0.003	0.003	0.204	0.169	0.030	0.005	0.025	0.001	0.037	0.004	0.002	0.125
16.A($n_N\pi^*$4)	0.001	0.001	0.117	0.078	0.120	0.002	0.170	0.001	0.209	0.006	0.003	0.078
17.T→A(CT1)	0.009	0.001	0.006	0.005	0.014	0.000	0.003	0.001	0.010	0.002	0.008	0.004
18.A($n_N\pi^*$5)	0.001	0.001	0.066	0.116	0.140	0.002	0.145	0.001	0.193	0.006	0.001	0.088
19.T→A(CT2)	0.004	0.004	0.017	0.017	0.011	0.001	0.050	0.011	0.008	0.005	0.003	0.018
20.A→T(CT2)	0.014	0.032	0.001	0.004	0.008	0.106	0.012	0.017	0.002	0.053	0.004	0.009
21.A→T(CT3)	0.003	0.016	0.006	0.008	0.000	0.216	0.001	0.017	0.001	0.002	0.008	0.005
22.A($\pi\pi^*$6)	0.004	0.003	0.171	0.152	0.045	0.002	0.100	0.003	0.067	0.009	0.003	0.225
23.T($n_O\pi^*$4)	0.053	0.236	0.003	0.006	0.001	0.011	0.001	0.173	0.000	0.036	0.092	0.006

	13	14	15	16	17	18	19	20	21	22	23
13.T($n_O\pi^*$3)	0.490										
14.A($\pi\pi^*$4)	0.004	0.232									
15.A($\pi\pi^*$5)	0.001	0.120	0.323								
16.A($n_N\pi^*$4)	0.001	0.092	0.073	0.270							
17.T→A(CT1)	0.001	0.007	0.003	0.005	0.459						
18.A($n_N\pi^*$5)	0.001	0.049	0.077	0.222	0.006	0.279					
19.T→A(CT2)	0.011	0.013	0.004	0.048	0.004	0.029	0.590				
20.A→T(CT2)	0.044	0.002	0.003	0.003	0.001	0.004	0.001	0.616			
21.A→T(CT3)	0.013	0.003	0.004	0.002	0.000	0.001	0.001	0.006	0.536		
22.A($\pi\pi^*$6)	0.002	0.227	0.069	0.076	0.003	0.098	0.019	0.014	0.001	0.231	
23.T($n_O\pi^*$4)	0.226	0.008	0.003	0.001	0.001	0.001	0.008	0.023	0.048	0.003	0.404

Table S21: Eigenvalues (eV) of A' adiabatic LVC states and corresponding normalised eigenvectors showing the contribution of the $\pi\pi^*$ and CT diabatic states from the 22-state FrD-LVC model of AT in C_s symmetry at the FC point. Parametrized by CAM-B3LYP with 6-31G(d) basis set.

Adiabatic states→	S_1	S_3	S_4	S_6	S_{10}	S_{11}	S_{12}	S_{14}	S_{15}	S_{17}	S_{21}	S_{22}
Eigenval.(eV)→ Eigenvec. ↓	5.325	5.458	5.567	6.139	6.643	6.728	6.810	7.073	7.133	7.275	7.417	7.486
1.T($\pi\pi^*$1)	0.976	-0.080	-0.170	0.005	-0.048	-0.049	0.058	0.033	0.013	0.014	-0.007	-0.003
3.A(L_a)	0.112	0.972	0.175	-0.016	-0.025	-0.058	-0.078	-0.017	0.016	0.009	0.004	0.001
4.A(L_b)	0.150	-0.183	0.959	0.120	0.060	0.005	0.032	0.062	-0.005	-0.000	-0.001	-0.011
6.A→T(CT1)	-0.026	0.033	-0.104	0.979	-0.112	-0.014	-0.019	-0.011	-0.008	0.002	-0.117	-0.004
10.T($\pi\pi^*$2)	0.049	-0.002	-0.061	0.093	0.929	-0.137	-0.297	-0.088	-0.042	-0.006	0.007	0.055
11.T($\pi\pi^*$3)	0.071	0.012	-0.000	0.010	-0.003	0.905	-0.412	0.006	0.030	0.009	0.035	0.045
12.A($\pi\pi^*$3)	-0.016	0.112	-0.040	0.054	0.332	0.385	0.844	0.092	0.021	-0.009	0.005	-0.041
14.A($\pi\pi^*$4)	-0.035	0.019	-0.054	0.007	0.053	-0.055	-0.114	0.975	0.107	-0.102	-0.000	-0.055
15.A($\pi\pi^*$5)	-0.009	-0.020	0.008	0.009	0.026	-0.033	-0.006	-0.116	0.991	-0.034	-0.017	-0.001
17.T→A(CT1)	-0.019	-0.006	-0.004	-0.000	0.015	-0.012	0.001	0.098	0.044	0.992	0.001	0.038
21.A→T(CT3)	0.001	-0.003	-0.011	0.115	-0.022	-0.036	0.011	-0.001	0.015	-0.002	0.992	0.005
22.A($\pi\pi^*$6)	-0.003	0.001	0.008	0.001	-0.035	-0.020	0.064	0.059	0.007	-0.044	-0.007	0.994

Table S22: Eigenvalues (eV) of A" adiabatic LVC states and corresponding normalised eigenvectors showing the contribution of the nπ^* diabatic states from the 22-state FrD-LVC model of AT in C_s symmetry at the FC point. Parameterized by CAM-B3LYP with 6-31G(d) basis set.

Adiabatic states→	S_2	S_5	S_7	S_8	S_9	S_{13}	S_{16}	S_{18}	S_{19}	S_{20}	S_{23}
Eigenval.(eV)→ Eigenvec. ↓	5.390	5.686	6.155	6.442	6.635	6.949	7.187	7.370	7.405	7.693	9.115
2.T($n_O\pi^*$1)	0.995	0.002	-0.014	0.009	0.001	-0.011	-0.002	-0.011	-0.089	-0.035	-0.004
5.A($n_N\pi^*$1)	-0.003	0.949	-0.142	-0.003	0.249	0.014	0.105	-0.067	0.020	0.005	-0.002
7.A($n_N\pi^*$2)	0.008	0.166	0.942	0.022	-0.121	-0.034	0.176	0.162	-0.062	-0.013	-0.071
8.T($n_O\pi^*$2)	-0.005	0.000	-0.021	0.995	-0.005	0.051	0.005	0.009	0.057	-0.054	0.015
9.A($n_N\pi^*$3)	0.001	-0.252	0.149	0.007	0.929	0.008	0.190	-0.118	0.011	0.000	-0.002
13.T($n_O\pi^*$3)	-0.032	-0.000	0.017	-0.043	-0.007	0.877	-0.024	-0.053	-0.332	-0.335	-0.024
16.A($n_N\pi^*$4)	-0.001	-0.082	-0.216	-0.011	-0.158	0.025	0.941	0.176	-0.033	0.000	-0.036
18.A($n_N\pi^*$5)	-0.000	0.022	-0.118	-0.003	0.184	0.014	-0.178	0.952	-0.107	-0.001	-0.024
19.T→A(CT2)	0.004	0.011	0.057	-0.015	-0.006	0.016	0.042	0.039	-0.021	-0.012	0.996
20.A→T(CT2)	0.093	-0.007	0.057	-0.051	-0.014	0.445	0.008	0.096	0.812	0.341	0.006
23.T($n_O\pi^*$4)	-0.008	0.000	-0.001	0.065	-0.001	0.164	-0.011	-0.052	-0.446	0.875	0.002

Table S23: Diabatic energies ($E^{\mathrm{D}}_{ii}(0)$) and electronic couplings ($E^{\mathrm{D}}_{ij}(0)$) of A' states from the FrD-LVC 22-state model of AT in C_{s} symmetry at ground state equilibrium geometry (eV).

STATE	T ($\pi\pi^*1$)	A (L_a)	A (L_b)	A→T (CT1)	T ($\pi\pi^*2$)	T ($\pi\pi^*3$)	A ($\pi\pi^*3$)	A ($\pi\pi^*4$)	A ($\pi\pi^*5$)	T→A (CT1)	A→T (CT3)	A ($\pi\pi^*6$)
1.T($\pi\pi^*1$)	5.347											
3.A(L_a)	-0.019	5.475										
4.A(L_b)	-0.035	0.007	5.577									
6.A→T(CT1)	0.016	-0.007	0.061	6.157								
10.T($\pi\pi^*2$)	-0.080	0.013	0.043	-0.051	6.655							
11.T($\pi\pi^*3$)	-0.099	-0.023	-0.014	-0.007	0.006	6.737						
12.A($\pi\pi^*3$)	0.033	-0.132	0.074	-0.040	-0.056	-0.030	6.761					
14.A($\pi\pi^*4$)	0.050	-0.012	0.092	-0.016	-0.037	0.008	0.018	7.065				
15.A($\pi\pi^*5$)	0.017	0.032	-0.018	-0.005	-0.015	0.013	0.007	0.006	7.131			
17.T→A(CT1)	0.035	0.017	0.009	0.000	-0.006	0.009	-0.003	-0.023	-0.005	7.272		
21.A→T(CT3)	-0.010	0.010	0.003	-0.148	-0.001	0.024	0.001	-0.003	-0.006	0.000	7.398	
22.A($\pi\pi^*6$)	0.004	-0.003	-0.015	-0.003	0.042	0.032	-0.023	-0.019	0.000	0.008	-0.001	7.480

Table S24: Diabatic energies ($E^{\mathrm{D}}_{ii}(0)$) and electronic couplings ($E^{\mathrm{D}}_{ij}(0)$) of A" states from the FrD-LVC 22-state model of AT in C_{s} symmetry at ground state equilibrium geometry (eV).

STATE	T ($n_O\pi^*1$)	A ($n_N\pi^*1$)	A ($n_N\pi^*2$)	T ($n_O\pi^*2$)	A ($n_N\pi^*3$)	T ($n_O\pi^*3$)	A ($n_N\pi^*4$)	A ($n_N\pi^*5$)	T→A (CT2)	A→T (CT2)	T ($n_O\pi^*4$)
2.T($n_O\pi^*1$)	5.410										
5.A($n_N\pi^*1$)	-0.001	5.780									
7.A($n_N\pi^*2$)	-0.001	-0.084	6.235								
8.T($n_O\pi^*2$)	0.003	-0.000	0.002	6.451							
9.A($n_N\pi^*3$)	-0.001	0.254	-0.024	0.000	6.594						
13.T($n_O\pi^*3$)	0.073	0.000	-0.000	0.026	-0.000	7.084					
16.A($n_N\pi^*4$)	-0.000	0.106	0.232	0.002	0.080	-0.000	7.124				
18.A($n_N\pi^*5$)	-0.001	-0.087	0.156	0.000	-0.088	-0.000	0.027	7.322			
19.T→A(CT2)	-0.015	-0.013	-0.194	0.042	-0.008	-0.050	-0.056	-0.033	9.095		
20.A→T(CT2)	-0.186	0.023	-0.063	0.036	-0.005	-0.210	0.004	0.004	0.000	7.323	
23.T($n_O\pi^*4$)	0.008	-0.000	-0.000	-0.080	0.001	-0.148	0.001	-0.000	0.000	0.057	7.608

S3.5.3 Photoexcitation to A(L_b) and T($\pi\pi^*$)

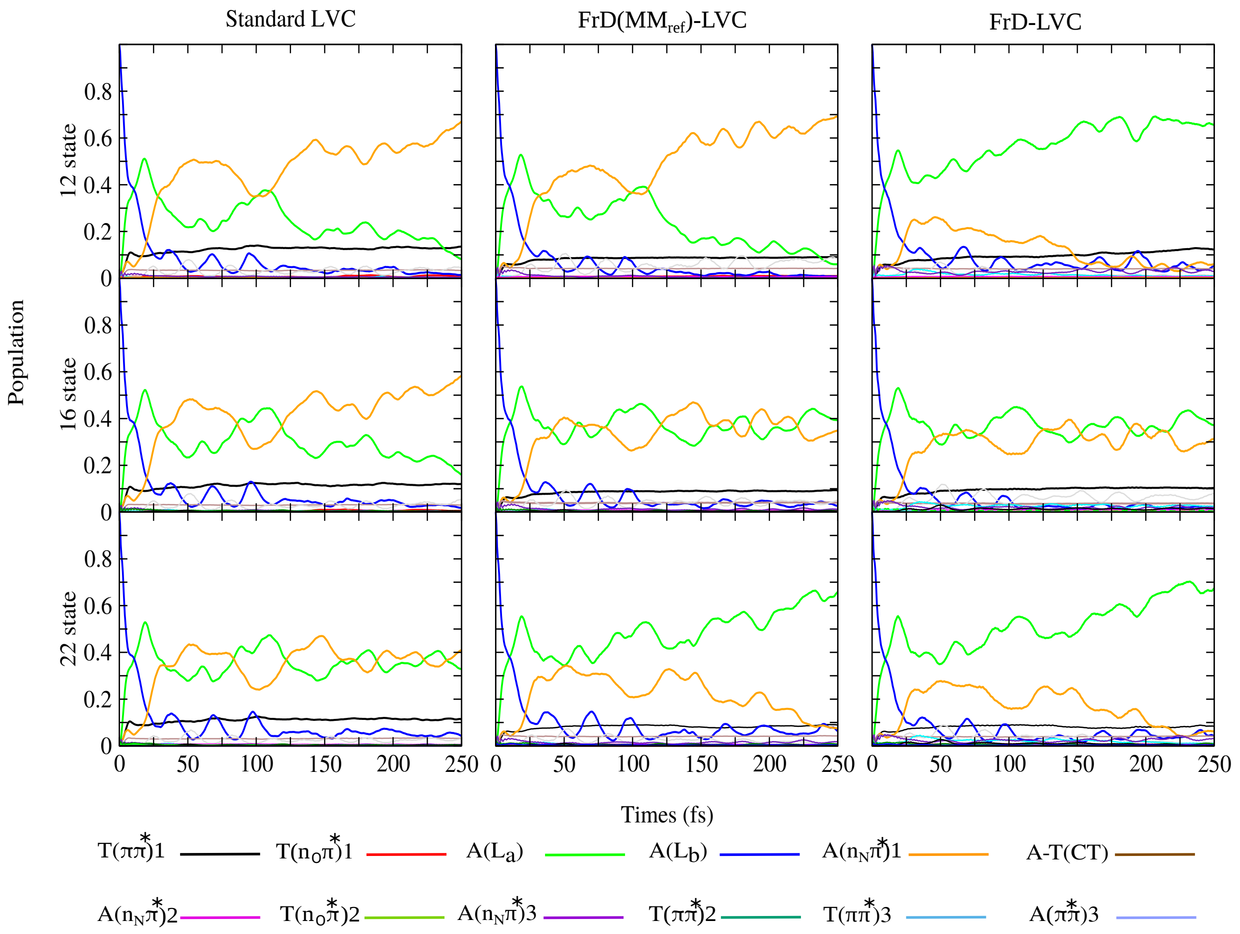

Figure S10: Diabatic state populations for AT following initial excitation of A(L_b) obtained with the st-LVC (left), FrD(MM_{ref})-LVC (middle) and FrD-LVC Hamiltonians (right) including different number of diabatic states.

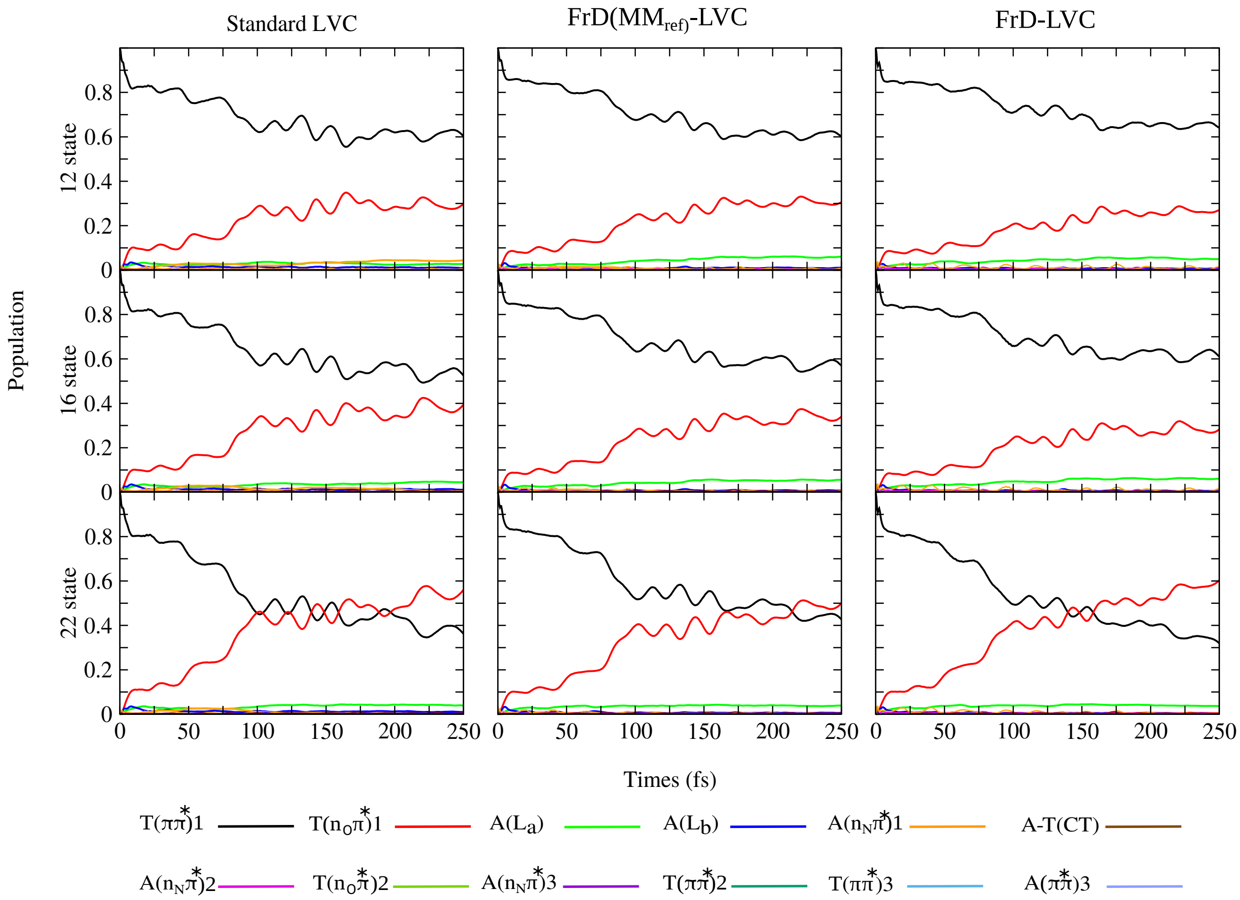

Figure S11: Diabatic state populations for AT following initial excitation of T($\pi\pi^*1$) obtained with the st-LVC (left) FrD(MM$_{ref}$)-LVC (middle), and FrD-LVC Hamiltonians (right) including different number of diabatic states.

S3.5.4 Effect of the increase of the number of diabatic states on isolated Adenine

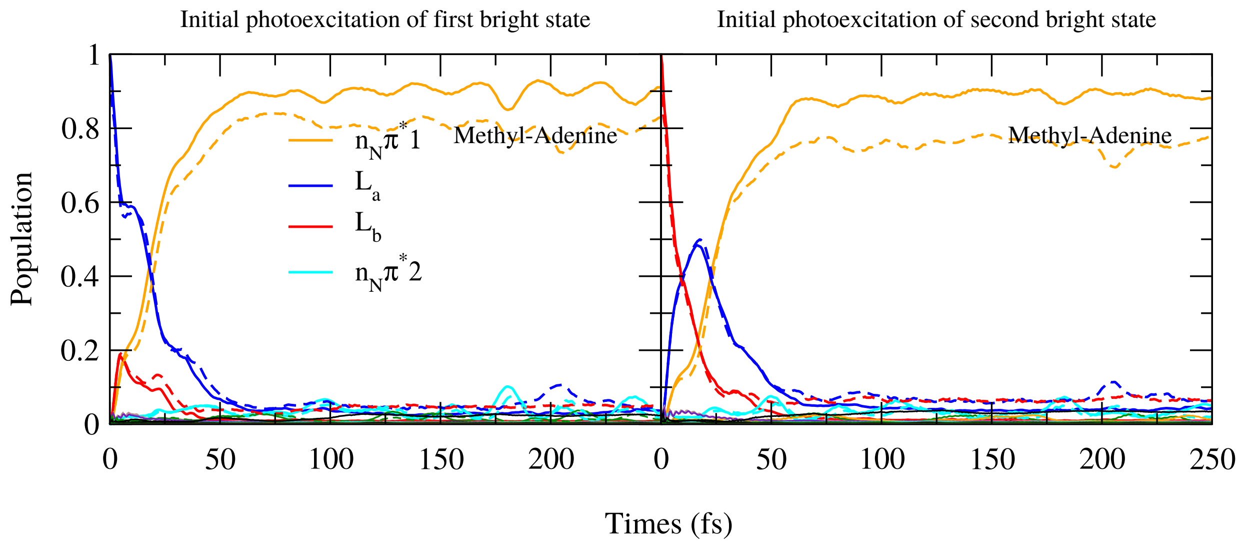

Figure S12: Diabatic state populations of 9methyl-adenine including either 6 (solid lines) or 15 (dashed lines) diabatic states. We checked that this larger model includes all the Adenine states we found to be important in either FrD-LVC or FrD(MM_{ref})-LVC calculations with 22 states. Initial excitation of the first bright state A(L_a) (left panel) and the second bright state A(L_b) (right panel). In particular state 12 affects the dynamics, although at the FC position it lies more than 2 eV higher in energy than the L_a and L_b states.

References

(S1) Liu, Y.; Cerezo, J.; Lin, N.; Zhao, X.; Improta, R.; Santoro, F. Comparison of the results of a mean-field mixed quantum/classical method with full quantum predictions for nonadiabatic dynamics: application to the $\pi\pi^*/n\pi^*$ decay of thymine. *Theor. Chem. Acc.* **2018**, *137*, 40.

(S2) Green, J. A.; Yaghoubi Jouybari, M.; Aranda, D.; Improta, R.; Santoro, F. Nonadiabatic Absorption Spectra and Ultrafast Dynamics of DNA and RNA Photoexcited Nucleobases. *Molecules* **2021**, *26*, 1743.

(S3) Improta, R.; Santoro, F.; Blancafort, L. Quantum mechanical studies on the photophysics and the photochemistry of nucleic acids and nucleobases. *Chem. Rev.* **2016**, *116*, 3540–3593.

(S4) Santoro, F.; Improta, R.; Fahleson, T.; Kauczor, J.; Norman, P.; Coriani, S. Relative Stability of the La and Lb Excited States in Adenine and Guanine: Direct Evidence from TD-DFT Calculations of MCD Spectra. *J. Phys. Chem. Lett.* **2014**, *5*, 1806–1811.

(S5) Green, J. A.; Yaghoubi Jouybari, M.; Asha, H.; Santoro, F.; Improta, R. A Fragment Diabatization Linear Vibronic Coupling Model for Quantum Dynamics of Multichromophoric Systems: Population of the Charge Transfer State in the Photoexcited Guanine Cytosine Pair. *Journal of Chemical Theory and Computation* **2021**, *submitted*.
